# Transcriptome Profiling and Molecular Pathway Analysis of Genes in Association with Salinity Adaptation in Nile Tilapia *Oreochromis niloticus*

**DOI:** 10.1371/journal.pone.0136506

**Published:** 2015-08-25

**Authors:** Zhixin Xu, Lei Gan, Tongyu Li, Chang Xu, Ke Chen, Xiaodan Wang, Jian G. Qin, Liqiao Chen, Erchao Li

**Affiliations:** 1 Laboratory of Aquaculture Nutrition and Environmental Health, School of Life Sciences, East China Normal University, 500 Dongchuan Rd., Shanghai 200241, China; 2 School of Biological Sciences, Flinders University, Adelaide, SA 5001, Australia; University Paris South, FRANCE

## Abstract

Nile tilapia *Oreochromis niloticus* is a freshwater fish but can tolerate a wide range of salinities. The mechanism of salinity adaptation at the molecular level was studied using RNA-Seq to explore the molecular pathways in fish exposed to 0, 8, or 16 (practical salinity unit, psu). Based on the change of gene expressions, the differential genes unions from freshwater to saline water were classified into three categories. In the constant change category (1), steroid biosynthesis, steroid hormone biosynthesis, fat digestion and absorption, complement and coagulation cascades were significantly affected by salinity indicating the pivotal roles of sterol-related pathways in response to salinity stress. In the change-then-stable category (2), ribosomes, oxidative phosphorylation, signaling pathways for peroxisome proliferator activated receptors, and fat digestion and absorption changed significantly with increasing salinity, showing sensitivity to salinity variation in the environment and a responding threshold to salinity change. In the stable-then-change category (3), protein export, protein processing in endoplasmic reticulum, tight junction, thyroid hormone synthesis, antigen processing and presentation, glycolysis/gluconeogenesis and glycosaminoglycan biosynthesis—keratan sulfate were the significantly changed pathways, suggesting that these pathways were less sensitive to salinity variation. This study reveals fundamental mechanism of the molecular response to salinity adaptation in *O*. *niloticus*, and provides a general guidance to understand saline acclimation in *O*. *niloticus*.

## Introduction

Salinity is one of the most significant factors regulating distribution, abundance and diversity of aquatic animals [1]. Due to diverse distributions of aquatic animals from freshwater to brackish or marine water, various physiological strategies have been evoluted for salinity adaptation [2, 3]. To reveal the adaptive processes of aquatic animals in salinity acclimatization, most studies have focused on growth [3], distribution [4], osmoregulation [5], production [6] and physiological responses [7, 8]. However, the underlying mechanism of salinity adaption in fish has not been well understood especially at the molecular level such as integrated molecular pathway responses. The existing literature in molecular biology is limited to specific gene cloning, function determination and biological pathways [9, 10], and little is known on the overall responsive pathways relevant to adaptive mechanism in fish to salinity changes.

*Oreochromis niloticus* is a unique model species to study salinity adaptation as it can live in a wide range of salinities [11, 12]. In the past decade, research on *O*. *niloticus* in brackish water has been confined to the influence of salinity on physiology, development and breeding [13–15]. Recently, studies in molecular biology have examined the influence of salinity on the expression of target genes such as the mRNA expression of Na^+^, K^+^-ATPase [16], renin-angiotensin system genes [17], transient receptor potential vanilloid 4 [18], growth hormone and somatolactin [19]. These fragmental studies have provided a basis to further explore adaptable strategies of *O*. *niloticus* to saline water. Therefore, there is a need to further study saline acclimatization of *O*. *niloticus* at a transcriptional level to reveal more fundamental mechanism in osmoregulation.

In fish, hepatopancreas is an important organ in energy metabolism [20] and detoxification [21, 22]. A recent study shows that hepatopancreas can help maintain salt and fluid balance under salinity chanlenge in Senegalese sole (*Solea senegalensis*) [23]. Salinity challenge adds more stress on aquatic animals [3], and therefore more energy is needed to maintain homeostasis [24]. As hepatopancreas plays mutiple functions in fish, it is an ideal organ to test its reponse to salinty challenge through a comprehensive molecular approach.

With the rapid advances in molecular technologies, it has become possible to explore the ecological and physiological mechanisms regulating distribution and function of aquatic organisms [25]. Multiple approaches using transcriptome, digital gene expression and proteomics have been developed to further understand the molecular change in cells and tissues of fish under salinity challenge [26, 27]. The transcription profiling method has been used to investigate the change of reference genes under environmental stress [26, 28]. The transcriptomic analysis is essential to explain the underlying functional elements of the genome and can lay a foundation to help understand the ability of an organism to cope with various stress [29]. With the emergence of transcriptome sequencing, RNA-Seq has significantly improved the gene coverage and increased the sensitivity for differentially expressed genes [30].

Therefore, the present study used RNA-Seq to reveal the hepatopancreas transcriptome differences of *O*. *niloticus* at different salinities. The pathways and genes that respond to salinity were obtained and analyzed. The results will provide an insight into the underlying mechanism of salinity acclimation in *O*. *niloticus* and its homologous species.

## Results

### Characteristics of the RNA-seq data

A total of 83.7 million reads were obtained, including 25.3 million reads in 0 practical salinity unit (psu), 27.7 million in 8 psu and 30.7 million in16 psu. After filtration, a total of 70.6 (81.4%) million reads (average length = 110 bp) were generated for subsequent analysis, including 25.1 (90.6%) million reads in 0 psu, 22.6 (81.2%) million in 8 psu and 22.9 (81.8%) million in 16 psu. The unique mapping reads were 19.2 (83.9%) million in 0 psu, 18.4 (81.5%) million in 8 psu and 20.6 (82.0%) million in 16 psu (Table 1).

**Table 1:** Summary statistics of the RNA-seq data.

Sample Name	0‰	8‰	16‰	Total	Average
Total reads (×10^6^)	25.3	27.7	30.7	83.7	27.9
Total reads after (×10^6^)	22.9	22.6	25.1	70.6	23.5
Reads filter (%)	90.6	81.7	81.8	84.3	84.2
Mapped reads (×10^6^)	19.6	18.8	20.9	59.3	19.7
Mapping rate (%)	85.5	83.0	83.5	84.0	83.8
Unique mapping (×10^6^)	19.2	18.4	20.6	58.2	19.4
Unique mapping rate (%)	83.9	81.5	82.0	82.4	82.6

### Differentially expressed genes and series clusters

**Fig 1:**
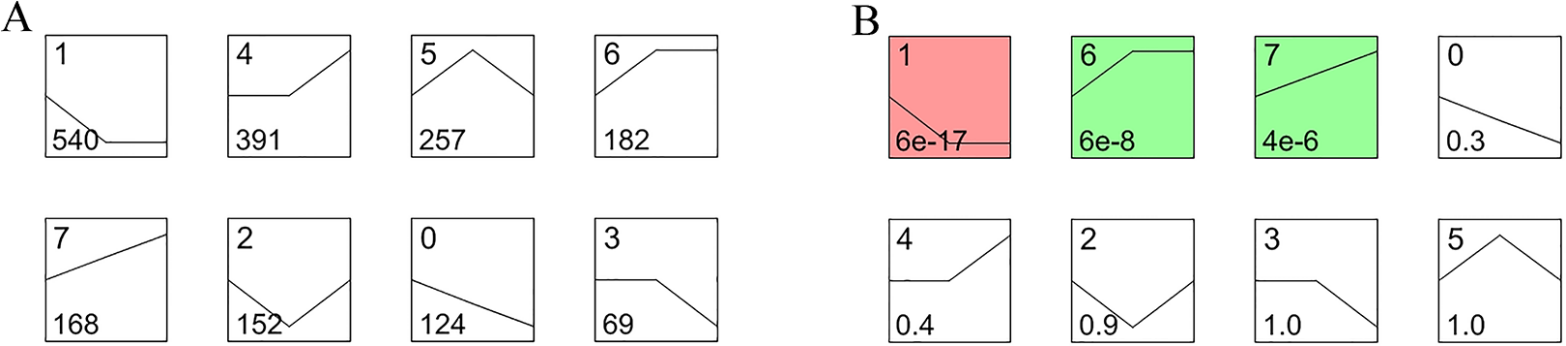
Eight types of gene change tendencies signed with gene numbers. (A): Numbers cited at the upper left corner represent each tendency. Figures cited at the lower left corner represent the number of genes in the tendency. (B): Numbers cited at the upper left corner represent each tendency. Numbers marked at the lower left corner represent the P-value of each tendency type. The colors represent significantly different levels.

The identification of genes was based on the Nile tilapia genome. To provide the gene annotation, the nucleic acid sequences of the genes were compared to the genomes of zebrafish, human, mouse and rat based on the amiGo database. Finally, a total of 296,000 genes were annotated. A total of 934, 1087 and 734 genes were differently expressed with either a fold change >2 or a fold change <0.5 (P < 0.05, FDR < 0.05) in the 0 vs 8 psu set, 0 vs16 psu set and 8 vs 16 psu set, respectively. Based on the comparison among these three groups, we obtained 1852 genes in differential gene unions and conducted eight types of unique model expression tendencies according to the amount of mRNA in relevant genes (Fig 1A and 1B). Tendencies 1, 6 and 7 were significantly different as calculated with Fisher’s exact test and the multiple comparison tests (P < 0.05). Tendency 1 contained the most differentially expressed genes. Because tendency 2 and tendency 5 shared the same expressed genes without difference between the freshwater control and 16 psu, the analysis of these two tendencies was meaningless and not conducted.

### GO and Pathway analysis

Gene ontology (GO) analysis involved annotated genes from functionally known species, and the gene products were divided into three categories: molecular function, cellular component and biological process. The unions of significantly enriched GO terms (P < 0.05) under biological processes containing three groups were divided according to different tendencies. There were 229 GO terms in tendency 0, 210 terms in tendency 1, 145 terms in tendency 3, 265 terms in tendency 4, 122 terms in tendency 6, and 152 terms in tendency 7 (Table 2).

**Table 2:** GO terms and GO pathways (P<0.05) with all differently expressed genes.

Tendency	0	1	3	4	6	7
GO terms	229	210	145	265	122	152
Different genes	107	453	51	324	139	132
KEGG pathways	7	19	4	26	10	10
Different genes	46	213	18	161	44	59

The Kyoto encyclopedia of genes and genomes database was used to obtain significantly changed pathways containing differentially expressed genes. We divided the 6 tendencies into 3 categories (Tables 3–5): constant change (containing tendencies 0 and 7), change-then-stable (containing tendencies 1 and 6) and stable-then-change (containing tendencies 3 and 4). Because tendencies 2 and 5 had no difference between the freshwater control and 16 psu, these pathways were not further analyzed. In the constant-change category, the complement and coagulation cascades contained four genes; the steroid hormone biosynthesis, steroid biosynthesis, and ovarian steroidogenesis contained 12 genes; and fat digestion and absorption, vitamin digestion and absorption and retinol metabolism contained eight genes. The stable-then-change category contained 31 pathways such as the biosynthesis of unsaturated fatty acids, fatty acid elongation, protein processing in endoplasmic reticulum, glycolysis/gluconeogenesis, pyruvate metabolism and tight junction. The change-then-stable category involved 29 pathways related to lipid metabolism, cell cycle and oxidative phosphorylation.

**Table 3:** Pathways contained in the constant change category.

Tendency	Pathways	P-value	Differentially expressed genes
0	Fat digestion and absorption	6.06E-03	APOA1, DGAT2, MOGAT2
	Retinol metabolism	1.78E-02	UGT2A1, DGAT2, DHRS13
	Vitamin digestion and absorption	2.88E-02	RBP2, APOA1
	Alzheimer's disease	2.72E-03	LRP1,MAPT,NDUFA4,CAPN2, CASP7, NDUFA7
	Parkinson's disease	1.51E-02	SLC6A3, VDAC3, NDUFA4, NDUFA7
	Chemical carcinogenesis	1.74E-02	UGT2A1, CBR1, GSTT3
	Metabolism of xenobiotics by cytochrome P450	1.70E-02	GSTT3, UGT2A1, CBR1
7	Steroid hormone biosynthesis	2.69E-03	UGT2A1,SULT3A1, SULT2B1, HSD17B7
	Steroid biosynthesis	2.35E-06	HSD17B7, LSS, SQLE, FDFT1, DHCR24
	Ovarian steroidogenesis	3.21E-02	CYP2J6, ADCY8, HSD17B7
	Sulfur metabolism	1.55E-02	SULT3A1, SULT2B1
	Arginine and proline metabolism	1.73E-02	OTC, ODC1, SAT1
	Protein digestion and absorption	3.50E-02	COL5A1, COL11A1,COL17A1, SLC7A9
	Porphyrin and chlorophyll metabolism	4.92E-02	UGT2A1, CP
	Complement and coagulation cascades	1.01E-02	C6, C3, PLG, SERPINF2

**Table 4:** Pathways contained in the change-then-stable category.

Tendency	Pathways	P-value	Differentially expressed genes
1	Fat digestion and absorption	7.04E-03	PPARA,PPAP2A,FABP2, PLA2G12B, APOB, APOA1
	PPAR signaling pathway	1.68E-04	APOA1, FABP4, ACSL5, PPARA, APOA1, FABP6, ACSBG2, FABP2, FABP4, RXRG
	Ether lipid metabolism	3.77E-02	PPAP2A, PLD1, PLA2G12B, PLB1
	Metabolic pathways	1.73E-04	NDUFS4, NDUFS5, PLB1, ALPL, CYP4F18, FUT9, GOT1, RFK, GGT5, LCT, POLE4, ASAH2, NDUFS1, COX6A1, NME3, CYP2R1, CYP27B1, COX5A, NDUFS6, ALPI, INPP5J, UCK2, NDUFB2, COX7C, GCDH, LCT, PGP, ATP6V1H, SAT2, GCH1, FASN, UQCRQ, LCMT2, NDUFA11, ACLY, ACSL5, NDUFB3, ACLY, COX6B1, NDUFA4, POLR2J, THTPA, PPAP2A, PLD1, UROD, ACSBG2, LIPASE, DBH, COX7B, ACACA, FBP2, ODC1, NT5C2, PHOSPHO1, LPIN1, GDA, URAH, COX6B1, LIAS, DCTD, GCK, PLA2G12B, DTYMK
	Fatty acid biosynthesis	2.36E-02	FASN, ACACA
	Ribosome	4.42E-14	RPS27L, RPS25, RPS15A, RPL28, RPS27A, RPS27RT, MRPS16, RPS12, RPS29, RPL26, RPS23, MRPS17, MRPS21, RPS27RT, RPL32, RPL35, RPS5, RPL13A, RPL37, RPL17, RPS10, RPL35A, RPL7A, RPS28, RPS19
	Proteasome	3.37E-02	PSMB6, SHFM1, PSMB4, PSMB9
	Vitamin digestion and absorption	3.37E-02	APOA1, APOA1, PLB1, APOB
	Folate biosynthesis	1.38E-02	ALPI, GCH1, ALPL
	Oxidative phosphorylation	3.31E-07	COX7A2, NDUFS1, COX6A1, COX5A, NDUFS6, NDUFB2, COX7C, ATP6V1H, UQCRQ, NDUFA11, NDUFB3, COX6B1, NDUFA4, COX7B, COX6B1, NDUFS4, NDUFS5
	Alzheimer's disease	8.00E-05	NDUFS4, NDUFS5, ERN1, COX7A2, NDUFS1, COX6A1,COX5A,NDUFS6, NDUFB2, COX7C, APOA1, UQCRQ, NDUFA11, NDUFB3, COX6B1, NDUFA4, COX6B1, COX7B
	Parkinson's disease	1.38E-05	COX6B1,NDUFS4,NDUFS5,COX5A, COX7A2,NDUFS1,COX6A1,UQCRQ, NDUFS6,NDUFB2,COX7C, COX7B, NDUFA11, NDUFB3, COX6B1, NDUFA4
	Non-alcoholic fatty liver disease (NAFLD)	1.36E-06	COX7B,COX6B1,NDUFS4,ERN1,NDUFS5,JUN, COX7A2, COX6A1, COX5A, NDUFS6, NDUFB2, COX7C, UQCRQ, NDUFA11, NDUFB3, COX6B1, NDUFA4, PPARA
	Huntington's disease	1.85E-04	COX6B1, NDUFA4, POLR2J, COX7B, COX6B1, NDUFS4, NDUFS5, COX7A2, NDUFS1, COX6A1, COX5A, NDUFS6, NDUFB2, COX7C, UQCRQ, NDUFA11, NDUFB3
	Cardiac muscle contraction	3.89E-06	ACTC1, COX6B1, COX7B, COX6B1, TNNC2, COX7A2, COX6A1, COX5A, TPM1, COX7C, MYH6, ATP1B1, MYL4, ACTC1, TNNT2, UQCRQ
	Dilated cardiomyopathy	4.86E-03	TNNT2, ITGA6, ACTC1, TNNC2, IGHV9, DES, ITGB4, TPM1, ITGB3, MYH6, ITGA6, ACTC1
	Hypertrophic cardiomyopathy (HCM)	7.40E-03	ITGA6, ACTC1, TNNT2, ITGA6, ACTC1, TNNC2, DES, ITGB4, TPM1, ITGB3, MYH6
	Small cell lung cancer	1.36E-02	RXRG,FHIT, RXRG,FHIT, TRAF5, ITGA6, CDK4, CDK4, ITGA6, LAMC3, MYC
	Pyrimidine metabolism	2.88E-02	POLE4, NME3, UCK2, POLR2J, NT5C2, DCTD, DTYMK
6	MicroRNAs in cancer	2.39E-03	CDKN1B, SOX12, CYP24A1, CD44, BMF, FSCN1
	Circadian rhythm—mammal	2.85E-02	PRKAG3
	Rheumatoid arthritis	3.20E-02	ANGPT1, H2-EA-PS, H2-EB2
	Asthma	3.55E-02	H2-EB2, H2-EA-PS
	Staphylococcus aureus infection	2.67E-02	H2-EA-PS, H2-EB2, ITGAX
	Cell cycle	4.97E-02	CDKN1B,STAG1,MCM6
	Purine metabolism	3.10E-02	NT5C2, ENTPD2, PDE1B, PDE6D
	Hematopoietic cell lineage	3.38E-02	ITGAX, CD44, H2-EB2
	Leishmaniasis	3.85E-02	ITGAX, H2-EA-PS, H2-EB2
	ECM-receptor interaction	3.85E-02	COL6A6, CD44, ITGA11

**Table 5:** Pathways contained in the stable-then-change category.

Tendency	Pathways	P-value	Differentially expressed genes
3	Retinol metabolism	2.09E-02	BCMO1,UGT1A5
	Folate biosynthesis	4.11E-02	ALPL
	Other types of O-glycan biosynthesis	1.02E-02	B4GALT1, UGT1A5
	Glycosaminoglycan biosynthesis—keratan sulfate	2.39E-03	B3GNT7, B4GALT1
4	Biosynthesis of unsaturated fatty acids	3.18E-02	ACOT7, ACOT1, ACOT3
	Metabolic pathways	4.55E-02	CYP3A25,ALOX5,ACSS1,CYP3A1,PRODH,LPIN1,GAL3ST1,TPI1, CKM,CBR1,PKM,CYP17A1,NME2,POLR2H,PGAM2,AK5,CKMT2,GFPT2,CKM,DHODH,PCYT1B,LAO1, PCK1,LDHA,NDUFV1,PTGDS, PISD,ALDOA,IMPA1,GLS,GADL1, PIGV,XYLT1,IDH2,GADL1,AMPD, HK1, PHOSPHO1
	Thyroid hormone synthesis	1.40E-05	HSPA5,HSPA5,HSPA5,PDIA4, HSPA5,HSPA5,HSPA5,HSP90B1, ATP1A3,HSPA5,HSPA5
	Ovarian steroidogenesis	3.96E-02	ACOT3,ALOX5, CYP17A1, ACOT1, IGF1R
	Protein processing in endoplasmic reticulum	4.99E-07	HSPA5, PDIA4, CALR, HSPA5, HSPA5, HSPA5, HSP90B1, HSPA5, PDIA6, HSPA5, HYOU1, DNAJB11, HSPA5, SEC31A, DERL1, CALR, HSPA5, CALR, SSR1
	Arginine and proline metabolism	1.64E-02	CKM, GLS, PRODH, CKM, CKMT2
	Protein export	2.30E-07	HSPA5, HSPA5, HSPA5, HSPA5, HSPA5, HSPA5, HSPA5, HSPA5
	Biosynthesis of amino acids	3.23E-02	PGAM2, ALDOA, IDH2, TPI1, PKM
	Glycolysis/gluconeogenesis	1.89E-04	PKM, PGAM2, PCK1, LDHA, ALDOA, HK1, ACSS1, TPI1
	Carbon metabolism	1.57E-02	TPI1, PKM, PGAM2, ALDOA, IDH2, HK1, ACSS1
	Proximal tubule bicarbonate reclamation	1.59E-02	SLC9A3, PCK1, GLS, ATP1A3
	Pyruvate metabolism	2.10E-02	ACSS1, PKM, PCK1, LDHA
	Chemical carcinogenesis	3.96E-02	GSTM5, CYP3A25, CYP3A13, CBR1, GSTO1
	Metabolism of xenobiotics by cytochrome P450	3.83E-02	GSTO1, GSTM5, CYP3A25, CYP3A13, CBR1
	p53 signaling pathway	3.12E-02	GADD45G,VAT1L,IGFBP3,GADD45B,ZMAT3
	PI3K-Akt signaling pathway	3.39E-02	DDIT4,VAT1L, IGFBP3, GADD45B, ZMAT3
	Cardiac muscle contraction	3.00E-03	MYH6,RYR1,MYH7,ATP2A1,MYH6, TPM1, TNNC2, ACTC1, ATP1A3
	Dilated cardiomyopathy	5.50E-04	ACTC1, MYH6, RYR1, MYH7, TTN, TITIN, ATP2A1, MYH6, TPM1, TNNC2, ACTB, SGCG
	Hypertrophic cardiomyopathy (HCM)	2.97E-04	ACTB, SGCG, ACTC1, MYH6, RYR1, MYH7, TTN, TITIN, ATP2A1, MYH6, TPM1, TNNC2
	Prion disease	8.71E-07	HSPA5,HSPA5,HSPA5,HSPA5,C7,HSPA5,EGR1,HSPA5,HSPA5,HSPA5
	Viral myocarditis	9.13E-07	MYH4,MYH4,ACTB,SGCG,MYH6,MYH4, MYH13, MYH7, MYH2, MYH1, EIF4G3, MYH2, MYH4, MYH6, MYH4, MYH4, MYH4
	Antigen processing and presentation	2.65E-05	CALR, HSPA5, HSPA5, HSPA5, HSPA5, HSPA5, HSPA5, CALR, HSPA5, CALR, HSPA5
	Chagas disease	3.32E-02	CALR, GNA14, CALR, IRAK1, JUN, CALR, TLR13
	Arrhythmogenic right ventricular cardiomyopathy (ARVC)	4.57E-02	ACTB,SGCG,RYR1,ACTN3,ATP2A1,DSG2
	HIF-1 signaling pathway	2.08E-02	EGLN3, IGF1R, LDHA, CAMK2D, EPO, ALDOA, HK1
	Tight junction	1.37E-06	MYH6, CLDN4, MYH4, MYH13, MYH7, ACTN3, MYH2, MYH1, MYH2, MYLPF, MYH4, MYH6, MYH4, MYH4, MYH4, MYH4, MYH4, ACTB, CLDN4, MYLPF

### Gene-act network and co-expression network analysis

**Fig 2:**
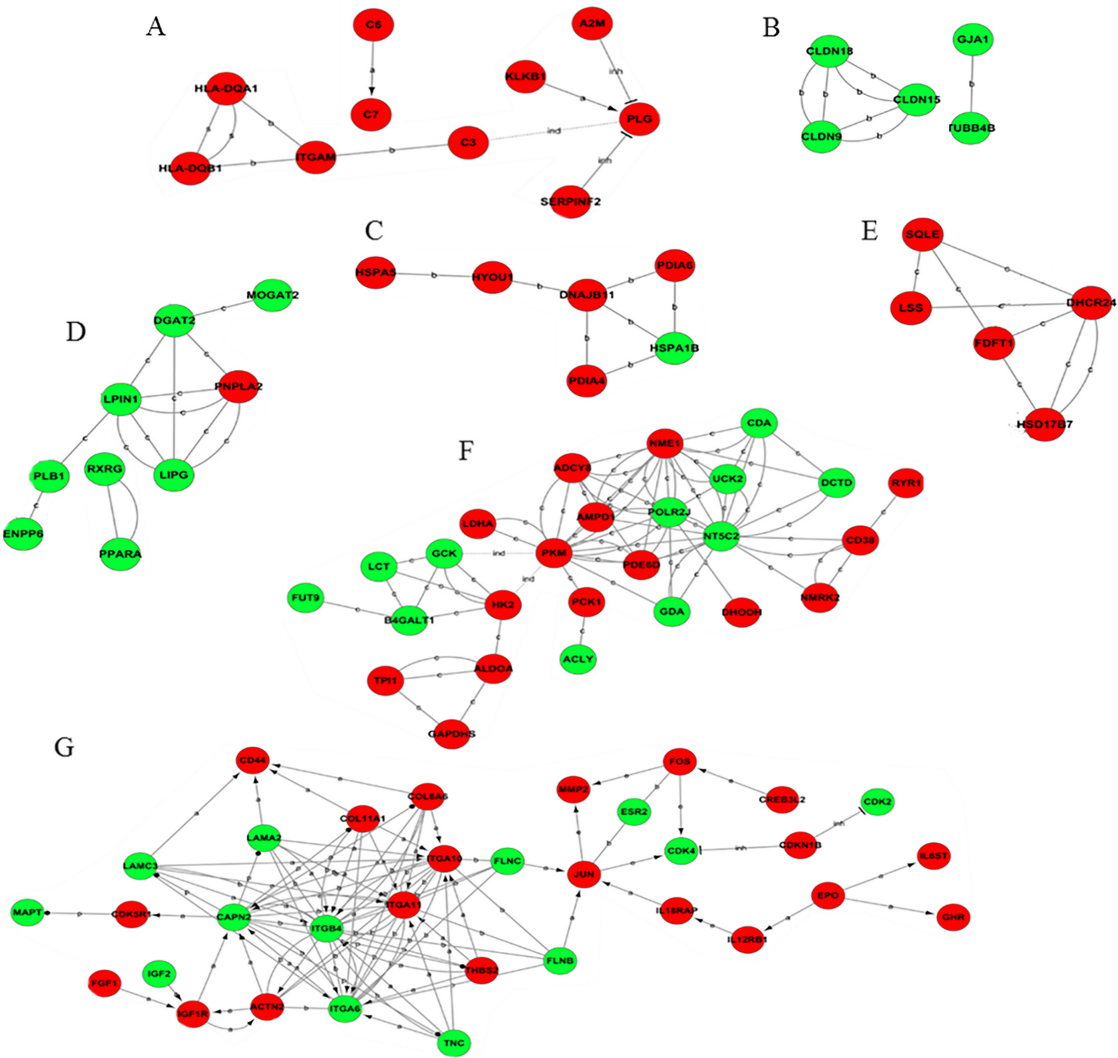
Gene act subnetworks in the 0 vs 16 psu categories. (A): complement and coagulation cascades; (B): cell connection; (C): endoplasmic reticulum activity; (D): fat digestion and absorption; (E): steroid biosynthesis; (F): glycolysis/gluconeogenesis; (G): signal transport. The red ball represents the up-regulated genes, while the green ball represents the down-regulated genes. The connections of genes were generated from the data analysis comprising GO analysis and KEGG pathway. The solid lines represent the relationships between genes. The dashed lines represent the genes that have an indirect effect. The arrow represents activation. The flathead represents suppression. ‘a’ represents activation; ‘b’ represents binding; and ‘c’ represents compound.

A total of 162 differentially expressed genes were involved in the constant change category, and they were used to build a gene-act network profile. A total of 72 genes were down-regulated, while 90 genes were up-regulated. The main subnetworks included fat digestion and absorption, glycolysis/gluconeogenesis, steroid biosynthesis, complement and coagulation cascades, endoplasmic reticulum activity, cell connection and signal transport (Fig 2A–2G).

**Fig 3:**
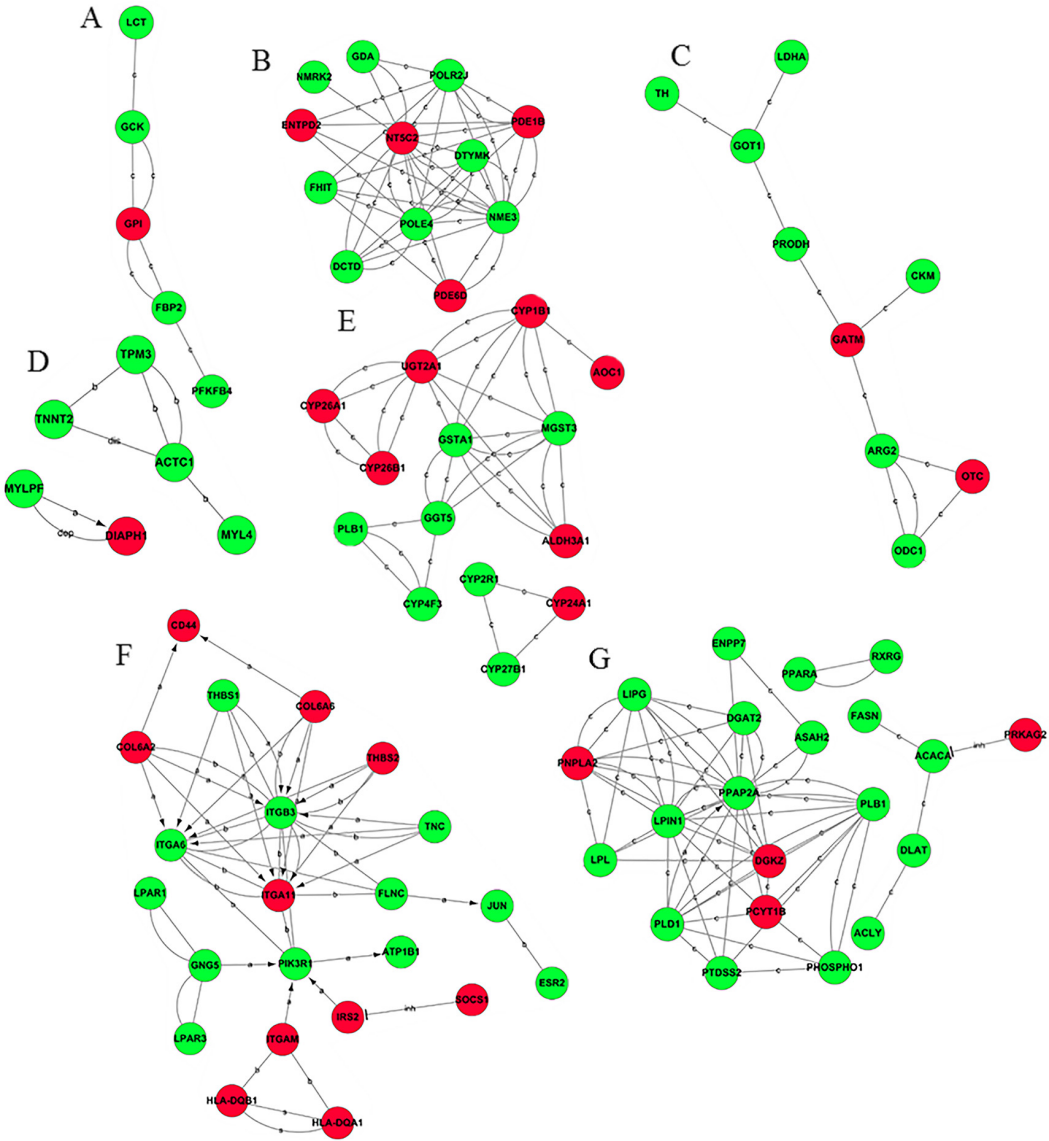
Gene act subnetworks in the 0 vs 8 psu categories. (A): glucose utilization; (B): nucleotide metabolism; (C): protein and amino acid metabolism; (D): cytoskeleton; (E): oxidative stress; (F): PI3K signaling pathways; (G): lipid and glycerophospholipid metabolism. The red ball represents the up-regulated genes, while the green ball represents the down-regulated genes. The connections of genes were generated from the data analysis consisting of GO analysis and KEGG pathway. The solid lines represent the relationships between genes. The dashed lines represent the genes that have an indirect effect. The arrow represents activation. The flathead represents suppression. ‘a’ represents activation; ‘b’ represents binding; and ‘c’ represents compound. The abbreviation ‘dep’ represents phosphorylation; ‘dis’ represents dissociation; and ‘inh’ represents inhibition.

The gene-act network profile of the change-then-stable category was composed of 113 differentially expressed genes with 36 up-regulated genes and 77 down-regulated genes. The major subnetworks contained lipid and glycerophospholipid metabolism, glucose utilization, protein and amino acid metabolism, PI3K signaling pathways, nucleotide metabolism, oxidative stress and cytoskeleton (Fig 3A–3G).

**Fig 4:**
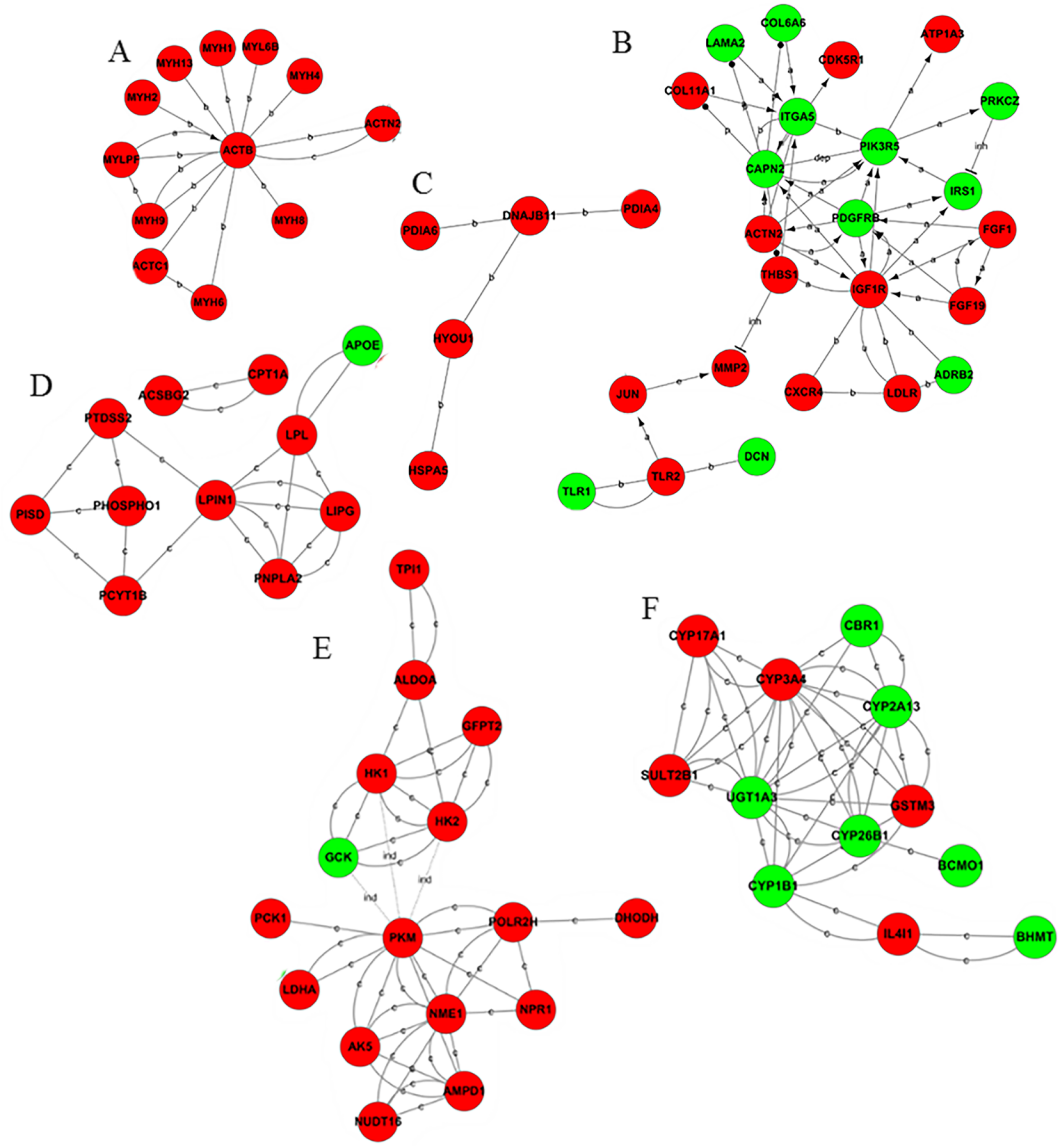
Gene act subnetworks in the 8 vs 16 psu categories. (A): cytoskeleton; (B): PI3K signaling pathways; (C): endoplasmic reticulum activity; (D): lipid and glycerophospholipid metabolism; (E): carbohydrates and pyruvate metabolism; and (F): steroid hormone metabolism and oxidative stress. The red balls represent up-regulated genes, while the green balls represent down-regulated genes. The connections of genes were generated from the data analysis consist of GO analysis and KEGG pathway. The solid lines represent the relationships between the genes. The dashed lines represent the genes that have an indirect effect. The arrow represents activation. The flathead represents suppression. ‘a’ represents activation; ‘b’ represents binding; and ‘c’ represents compound. The abbreviation ‘dep’ represents phosphorylation; ‘dis’ represents dissociation; and ‘inh’ represents inhibition.

A total of 101 differentially expressed genes composed the stable-then-change category, with 20 down-regulated genes and 81 up-regulated genes. The chief sub-networks which contained in this category were the signaling pathway of PI3K and p53, steroid hormone synthesis and oxidative stress, fat synthesis and glycerophospholipid metabolism, cytoskeleton, endoplasmic reticulum activity, sugar utilization and pyruvate metabolism (Fig 4A–4F).

**Fig 5:**
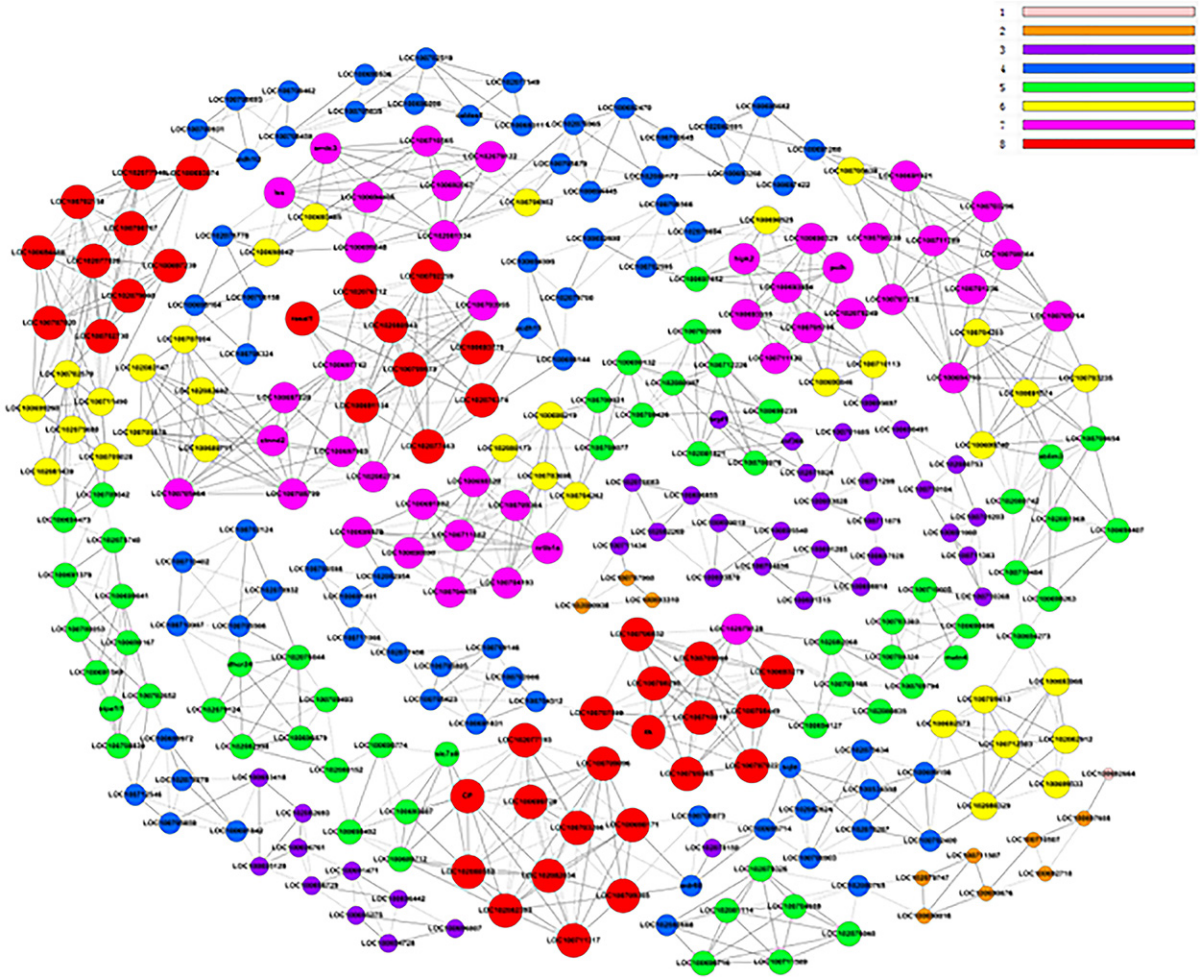
Co-expression network of differentially expressed genes in freshwater and 16 psu. The greater the value of k-core is, the more strongly the differentially expressed genes are co-expressed according to the size of the ball. The labels from one to eight represent the importance of genes. The red represents the most significant and the pink means the least significant.

The gene co-expression profile of the constant-change category contained 293 genes that were differentially expressed relative to others. To locate the core regulatory genes that were involved in the molecular response to salinity, core effect factors were determined by the degree differences between the freshwater and 16 psu (Fig 5). There were 40 genes in the red ball with 8 in the k-core, indicating that these genes would have the broadest contact with the surrounding genes and share the same expression tendencies under the same circumstances.

### Experimental validation

**Fig 6:**
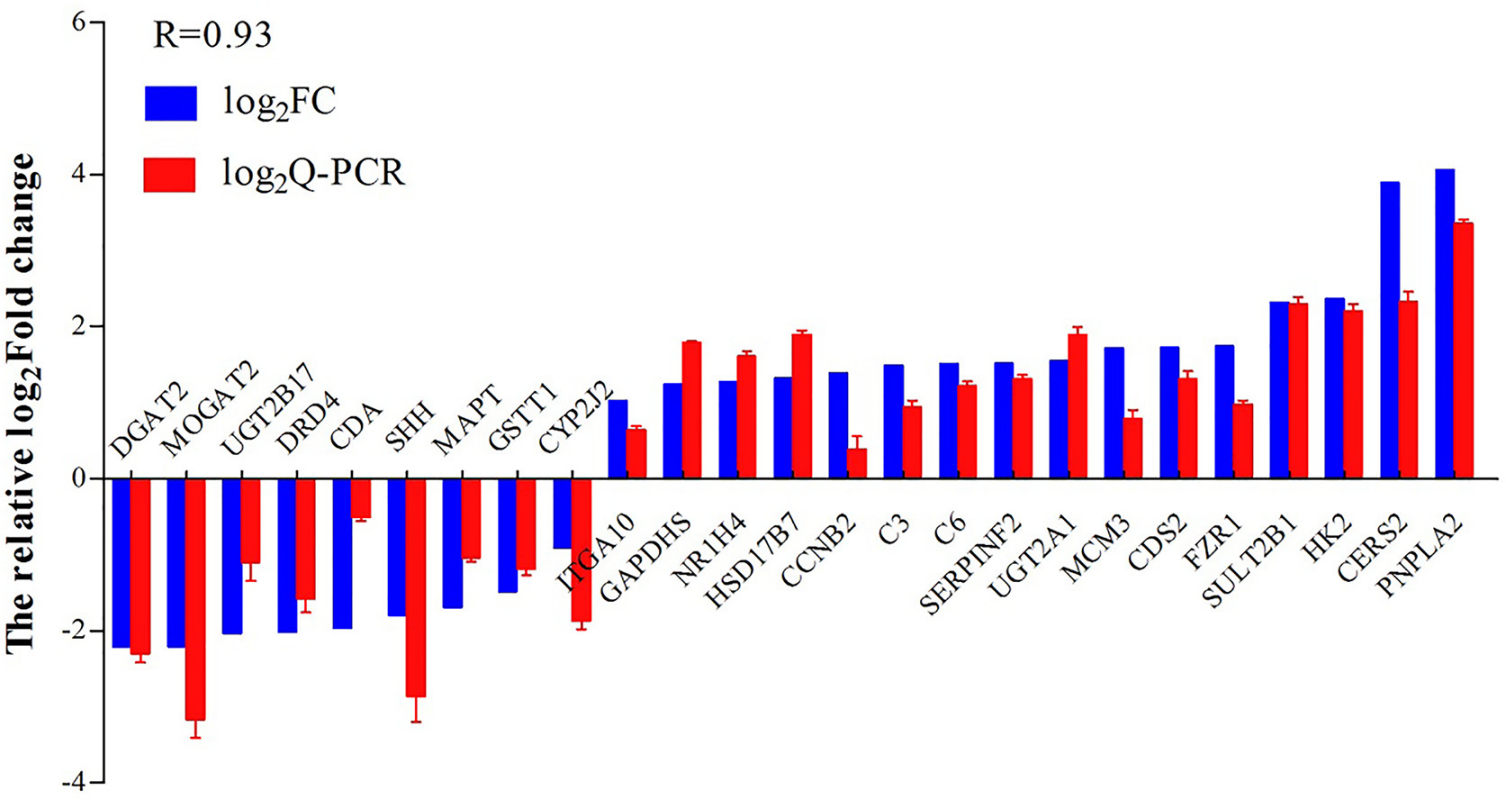
Validation of Q-PCR. Comparison of the relative log_2_ (fold changes) between RNA-Seq and qPCR after salinity acclimation compared to the control, as normalized with the EF1A gene.

Twenty five differentially expressed genes were randomly selected to validate the reliability of the RNA transcriptome in this study. Melting-curve analysis revealed a single product for all of the tested genes. Log_2_FCs from qPCR were compared with the results of RNA-Seq expression analysis (Fig 6). The results of Q-PCR and RNA-Seq showed a correlation coefficient of 0.93, indicating the credible RNA-Seq results.

## Discussion

### Pathway analysis in the constant-change category

The pathways in the constant-change category are sensitive to salinity fluctuation. When the pathways respond positively to salinity fluctuation, these intermediate products are required for the basic physiological activities [31]. Therefore, the stability maintenance of these pathways is primarily important in salinity acclimation of tilapia. Due to the sensitivity of these pathways, they may play critical roles in response to a variable environment, and it is imperative to conduct further research to alleviate the salinity stress on *O*. *niloticus* in a brackish water environment.

#### Steroid metabolism-related pathways

Steroid metabolism-related pathways such as steroid biosynthesis (S1 Fig), ovarian steroidogenesis (S2 Fig), sulfur metabolism and steroid hormone biosynthesis (S3 Fig) played pivotal roles in response to salinity stress and are inextricably linked to other pathways in aquatic animals [32, 33]. The steroid regulatory metabolism under salinity stress in *O*. *niloticus* is discussed below.

Biosynthesis of cholesterol is centered in the relationship between osmoregulation and steroid as the cholesterol induced by salinity is related to the physical properties of cell membranes [34] and steroid hormone in ovarian development. Steroid hormone demonstrates the osmoregulation ability in red blood cells against hypotonic hemolysis in dog [35, 36]. In the present study, in a saline environment, the ovarian steroidogenesis pathway activated the cAMP signal pathway and stimulated adenylate cyclase for cAMP-regulated gene transcription (S2 Fig) in tilapia hepatopancreas [37]. The cAMP signaling pathway stimulates the production of arachidonic acid metabolites to regulate hormone production, including cortisol and glucagon, osmoregulation and cellular fatty acid signaling in tilapia [37, 38].

The expression of gene sulfotransferase 2B1 promotes the production of cholesterol sulfate and dehydroepiandrosterone (DHEA) [39]. Previous research suggests that the organisms can utilize cholesterol sulfate to support platelet adhesion [40], regulate serine proteases to alter epidermal cell adhesion [41] and transmit with signal transduction by modulating selective protein kinase C isoforms and phosphatidylinositol 3-kinase (PI3K) [42, 43]. The changes in the PI3K signal pathway and cell adhesion in this study suggest that cholesterol sulfate affects salinity domestication in tilapia. Research on the relationship between DHEA and salinity adaptation is scarce. The DHEA could be indirectly affected by salinity domestication based on the present research. In other animal models, DHEA and its metabolites constitute α subunit activators, which is one class of peroxisome proliferators-activated receptors (PPAR) found in nature [44, 45] and also in the present study. The functions of DHEA, such as anti-inflammatory properties during inflammatory responses in mice [46], may be involved in the regulation of immune-related pathways. These findings could provide new research directions for studying DHEA in aquatic animals.

Combining the literature evidence with the RNA-Seq results in the present study, the sulfur metabolism response to salt adaptation is surmised nonspecifically by sulfonating pregnenolone, cholesterol and DHEA for efficiently regulating metabolism and steroids [47, 48].

#### Lipid metabolism-related pathways

Under salinity stress, fish must have appropriate mechanisms to regulate osmotic balance and to maintain basic physiological functions [49, 50] which has been verified in this study (S1 Table). It is reported that euryhaline fish and crab *Chasmagnathus granulata* can utilize lipids as an energy source when encountering osmotic stress [51, 52], which is similar to the results of the present study. In this experiment, *O*. *niloticus* might decrease triglyceride production and reserve lysophosphatidic acid, monoacylglycerols and fatty acids to spare energy for osmoregulation which is alike to the results of other study [53].

#### Immune-related pathways

Retinol metabolism, vitamin digestion and absorption, porphyrin and chlorophyll metabolism and the metabolism of xenobiotics by cytochrome P450 were gradually down-regulated while the complement and coagulation cascades were up-regulated by salt pressure in this study. The immune-related pathways in this category mainly cover the requirements of antioxidant and complement reactions that are altered by appropriate timing to salinity variation. The carotenoids (including β-carotene), retinol (vitamin A) and α-tocopherol (vitamin E), which are lipid-soluble antioxidants in aquatic animals [54], are utilized to contribute to antioxidants under salinity stress in the hepatopancreas. The transcripts of two genes encoding proteins involved in porphyrin and chlorophyll metabolism are changed and they can participate in oxidation-reduction reactions in freshwater fish [55]. In fish, the cytochrome P450 monooxygenase system metabolizes a large number of xenobiotic compounds to perform detoxification [56]. In addition, plasminogen, complement 3 and complement 6 [57] are highly expressed with salinity elevation to cope with anti-physiological challenges in the *O*. *niloticus* hepatopancreas.

#### Osmoregulation-related pathways

Constant salinity change provokes transcriptional up-regulation of genes involved in arginine and proline metabolism and protein digestion and absorption. In aquatic insect larvae, free amino acids such as arginine and proline act as regulators for osmoregulation [58]. In blue crab *Callinectes sapidus*, amino acids participate in cell volume regulation. However, the ways how these amino acids work in osmoregulation are not clear [59]. The arginine and proline metabolisms are also involved in transcriptome analysis of Chinese mitten crab under salinity stress [26]. Therefore, it is reasonable to deduce that constant salinity change provokes arginine and proline metabolisms which are helpful in conservative regulation under salinity stress in tilapia and other species.

### Pathway analysis in the change-then-stable category

The change-then-stable category displays pathways that are changed in tilapia transcriptome and that are sensitive to salt stress, but the changes would stop when the salinity concentration exceeds a threshold value. The reasons for maintaining stable stages in these pathways are possibly that (1) the compensation approaches can cover the environmental changes; and (2) the management of these pathways has reached a limit, implying that the condition may be stressful to the organism.

#### Cell activity-related pathway

The ribosome is most significantly changed in tendency 1 in this study. Ribosomal proteins are ubiquitous and abundant to bind to RNA, and participate in balancing the synthesis of the RNA and ribosome protein components [60]. The relationships between osmoregulation and ribosome in animals are little known, but the ribosome in plants can decrease under osmotic stress [61, 62] which is consistent with the present study and provides evolutionary evidence on the similarity between plants and animals for the ribosome in regulating osmotic stress. Changes in free ribosomal proteins in ribosome composition could have a relationship with the p53 system to regulate physiological activities [63], which also agrees with the result of the present study in the stable-then-change category. The cellular responses to osmotic stress affect the cell cycle and closely link to the DNA conformation and DNA activity [64]. Research on osmoregulation in chondrocytes has proven the reason for the change in the shape of the chondrocytes in the collagen fibrils [65]. This finding is consistent with the up-regulation of the collagen gene in the present study and shows that collagen participates in the salinity stress responses of the cells and tissues. These pathways could be involved in the regulatory mechanisms when an organism is under salt stress.

#### Immune-related pathways

Immune-related pathways are related to proteasome and oxidative phosphorylation with the characteristics of the change-then-stable category. In cells, the proteasome is inhibited by the p38 MAPK-dependent phosphorylation response to osmotic stress [66], which explains the down-regulation of proteasomes to alter the intracellular antigen process in the cytolytic immune response [67] under salt stress. The extracellular damage and change in osmotic pressure lead to an increase in the production of intracellular reactive oxygen species [68]. Therefore, extra reactive oxygen species can switch on as an immune response against homeostasis disorder.

#### Lipid metabolism-related pathways

The PPAR signaling pathway (S4 Fig), fat digestion and absorption (S5 Fig), metabolic pathways, fatty acid biosynthesis (S6 Fig) and ether lipid metabolism are significantly down-regulated by the change of osmotic pressure. According to the change of ATP under salinity stress in this study, acetyl-CoA is needed [69] to enable the reduction of fatty acid biosynthesis due to a lack of materials in the substrate. In response to fatty acid loss, steroid biosynthesis and the PPAR signaling pathway may be down-regulated to decrease apolipoprotein and the expression of long-chain acyl-CoA synthetase to activate fatty acid β oxidation [70]. The indirect evidence of the PPAR signaling pathway mediated by osmotic stress is that angiotensin II is a hormone response to osmotic stress as well as its association with the down-regulation of PPARs [71]. Ether lipid is an emerging class of lipids and its function in osmoregulation is an enigma, though they act a platelet-activating factor in the membrane components in the brain and testis [72].

### Pathway analysis in the stable-then-change category

Transcriptome differences among 8 psu, 16 psu and the freshwater environment are not sufficiently stressful to activate the pathways that are involved in the stable-then-change category. These regulatory pathways can adapt to a low salinity environment when the salinity reaches a threshold that these paths cannot withstand the change and result in functional adaptation at the transcriptional level [73, 74].

#### Energy metabolism-related pathways

The biosynthesis of unsaturated fatty acids (S7 Fig), ovarian steroidogenesis, fatty acid elongation, glycolysis, gluconeogenesis (S8 Fig) and pyruvate metabolism (S9 Fig) are significantly up-regulated in this study. The genes of acyl-coenzyme A thioesterase in the synthesis of unsaturated fatty acids are up-regulated at the transcriptional level to promote the synthesis of unsaturated fatty acids. This may be because *O*. *niloticus* needs to upregulate some fatty acids synthesis pathways to meet the demands for these fatty acids. Similarly, the high level of dietary n-3 highly unsaturated fatty acids improves the tolerance of Chinese mitten crab larvae to salinity stress [75]. It is well known that fish are liable to congenital diabetes due to a low ability of carbohydrate utilization [76]. During seawater acclimation in *Oreochromis mossambicus*, the glycogen content in the hepatopancreas is decreased significantly after transfer to seawater, suggesting that salt acclimation promotes carbohydrate utilization [77], which supports the findings in the current study. Thus, it is suggested that lipids are the main source of energy supply under salinity domestication in *O*. *niloticus*.

#### Protein metabolism-related pathways

Protein pathways, including thyroid hormone synthesis, protein processing in endoplasmic reticulum, arginine and proline metabolism, protein export and the biosynthesis of amino acids belong to tendency 4. In fish, thyroid hormone is an amino acid derivative that acts as a regulator for metabolism, osmoregulation and salinity adaptation [78, 79]. The thyroid hormone receptor regulates cholesterol and carbohydrate metabolism through direct actions on gene expression [80] as well as cross-talk with other nuclear receptors, including PPAR and the liver X receptor [81]. The result of this study suggests that material synthesis and transmission to counteract stress require a large amount of energy and related organelle activities.

#### Cell activity-related pathways

Tight junction and antigen processing and presentation are pathways of tendency 4, while other types of O-glycan biosynthesis and glycosaminoglycan biosynthesis-keratan sulfate belong to tendency 3. Long-term salinity acclimation leads to the increase of gene transcription in basic components of cytoskeleton such as tilapia claudins, actin and myosin, and the cytoskeleton can further interact with intercellular tight junctions [82]. The production of glycan in this study may play a role in information transmission due to its specificity in the immune response [83].

#### Signaling pathways

Signaling pathways such as the p53, PI3K-Akt and HIF-1 are increased by high salinity. P53 is a tumor suppressor protein and a critical component in cell cycle checkpoint response [84]. However, recent research suggests that the p53 level does not provoke an apparent increase in rainbow trout compared with mammals [85]. In aquatic animals, it is unclear whether p53 stimulated by salt adaptation is able to induce cancer cell proliferation. In this study, p53 transcription is not altered but the downstream genes are up-regulated, and there is no sufficient evidence to prove that salinity-induced carcinogenesis in tilapia. Insulin-like growth factor 1 receptor transcription is up-regulated to generate the PI3K-Akt signaling pathway and HIF-1 signaling pathway [86]. However, these conclusions in other animal models are not verified in aquatic animals.

## Conclusions

**Fig 7:**
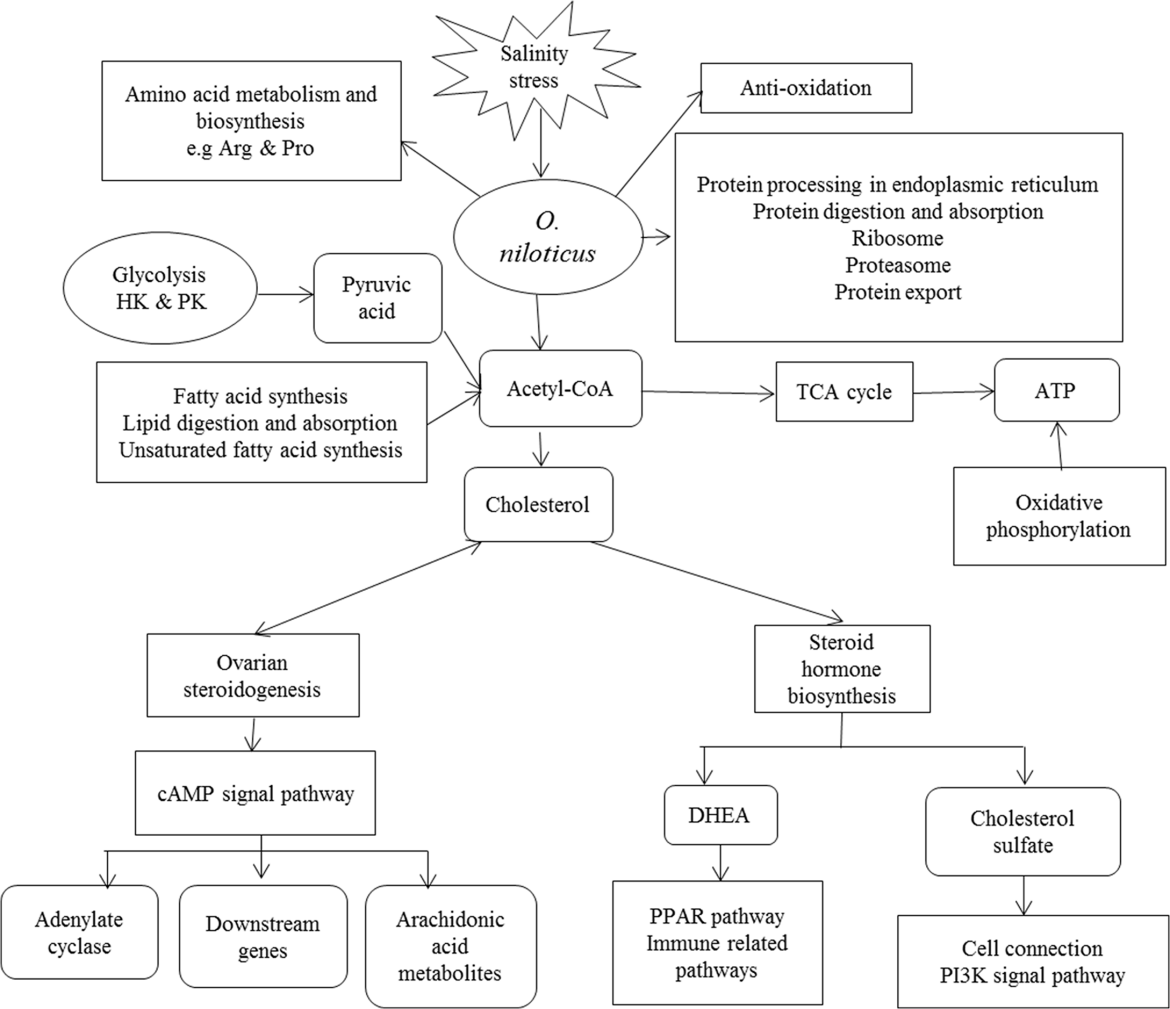
Summary of the transcriptional changes of *O*. *niloticus* is shown under the salinity domestication. The right-angle quadrilateral represents the pathways, and the rounded quadrilateral represents the important intermediates.

According to the survival and growth parameters of *O*. *niloticus* (S1 and S2 Tables), it is feasible to rear *O*. *niloticus* at saline water. From the molecular perspective as shown in Fig 7, during salinity acclimation, *O*. *niloticus* produced significant changes in amino acid metabolism and synthesis, oxidation, protein synthesis and degradation, energy material utilization, and signal transduction. Glycolysis and fatty acids are involved in the regulation of acetyl coenzyme A synthesis and metabolism to participate in the TCA cycle and produce ATP for energy supply. Acetyl coenzyme A also participates in cholesterol synthesis by adjusting the needs of ovarian steroids and steroid hormone synthesis. Ovarian steroidogenesis activates the cAMP signal pathway to regulate adenylate cyclase, downstream gene expression and arachidonic acid metabolites. Among these actions, adenylate cyclase catalyzes ATP into cAMP to support signal transmission and the downstream genes of cAMP signal pathway cover various physiological processes. Arachidonic acid metabolites play extensive roles in maintaining homeostasis. Steroid hormone biosynthesis produces cholesterol-containing DHEA and cholesterol sulfate, which in turn participate in the PPAR pathway, immune-related pathways, cell connections and the PI3K signal pathway. The synthesis and metabolism of some amino acids reflect the reaction of tilapia to maintain osmotic stability. Protein synthesis and the metabolism in organisms are a prerequisite before responding to an environmental salinity challenge. In short, the steroid hormones, osmoregulation, lipid metabolism and cell-connected components are critical measures for salinity domestication in aquatic animals.

## Materials and Methods

### Experimental design and sampling

*Oreochromis niloticus* juveniles (initial weight 0.88 ± 0.04 g) were purchased from a farm in Hainan, China. After acclimation in freshwater for two weeks, fish were stocked in nine freshwater tanks (66 × 63 × 40 cm) with 25 fish each. One set of three tanks with freshwater were used as control and other two sets of three tanks were acclimated to the salinities of 8 and 16 psu, respectively, by increasing the salinity at 4 psu per day using crystal sea salt. The salinity was daily measured with a salimeter (AZ Instrument Corp. Ltd, Co. AZ8371). Fish were exposed to three final salinities 0, 8, and 16 psu in triplicate for 8 weeks. During the experiment, the environment was maintained at 12:12 dark/light, 28 ± 1°C, 7.99 ± 0.23 pH and >6 mg/L dissolved oxygen. One third of the water volume was daily renewed with corresponding salinities. Tilapias were fed with a commercial diet (35% crude protein and 3% crude lipid, S3 Table) twice daily at 08:00 h and 16:00 h to apparent satiation. Feces and uneaten feed were daily removed with a siphon tube. At the end of the experiment, *O*. *niloticus* in all tanks were fasted for 24 h before sampling. All experiments were conducted under the standard code of protocol for the care and use of laboratory animals in China. This research project was approved by the Animal Ethics Committee of East China Normal University. Eight fish were randomly selected and were anesthetized in 30 ppm MS-222. The hepatopancreas of each fish was put in a sterile plastic tube with Trizol (Invitrogen) for RNA extraction.

### RNA extraction

The total RNA of the hepatopancreas was extracted by the Trizol method (Invitrogen). The integrity of every RNA sample was tested using 1% agarose gel electrophoresis to ensure that the RNA was integral with three distinct and bright stripes. In each treatment, the eight qualified RNA samples from each treatment group were mixed with the same amount of RNA to make sure the veracities and universalities. The quality of mixed RNA was further checked on a Bioanalyzer 2200 (Agilent Technologies, Santa Clara, CA, USA) with a RIN^e^ value of >8, which is acceptable for cDNA library construction (S10 Fig). The mRNA is purified using the Dynabeads mRNA Purification Kit (Life tech, Cat. no. 1264684).

### cDNA library construction and sequencing

The RNA-Seq was conducted by Novel Bioinformatics Co., Ltd with Sanger / Illumina 1.9. The cDNA library was prepared using the Ion Total RNA-Seq Kit v2 (Life technologies, Cat. no. 4479789) with 5 μg total RNA following the manufacturer's instructions. The mRNA with poly(A) was isolated with Dynabeads (Life technologies, USA), fragmented with RNaseIII and then purified. The fragmented RNA was added and ligated with ion adaptor. Double-stranded cDNA was synthesized and purified by the magnetic bead based method. The molar concentration of the purified cDNA was detected for each cDNA library. The filtering of the raw reads was produced with FAST-QC (http://www.bioinformatics.babraham.ac.uk/projects/fastqc/). Samples were processed on a OneTouch 2 instrument and enriched on a OneTouch 2 ES station for preparing the template-positive Ion PI Ion Sphere Particles according to the Ion PI Template OT2 200 Kit (Life tech, Cat. no. 4482286). After enrichment, the mixed template-positive Ion PI Ion Sphere particles of three samples were loaded onto a 1 P1v2 Proton Chip and sequenced on Proton Sequencers according to the Ion PI Sequencing 200 Kit (Life tech, Cat. no. 4482283).

### Quality control and mapping

The raw sequencing data were also evaluated by FAST-QC. The evaluation metrics scrutinized the nature of the data before subsequent variant evaluation. MapSplice software (MapSplice v2.1.8) was used, which is a tool for RNA-seq data analysis on mapping the RNA-seq data to the genome of *O*. *niloticus* Orenil1.1. MapSplice is the core program in Bowtie 1.0.0 that can quickly identify splicing and cutting the exon-exon [87]. The accession number of RNA-Seq is GSE67671.

### Differential expression and series cluster

Different gene expression was screened by the EB-Seq algorithm [88]. The differential gene screening criteria was fold change >2 or fold change <0.5 (P <0.05, and false positive rate (FDR) <0.05). If multiple transcripts existed in a gene, the longest transcript was selected to calculate the sequencing depth and expression to ensure the result accuracy. The whole differential gene union of the three sets was obtained. Any two of the three sets (0 vs 8, 0 vs 16 and 8 vs 16 psu) were compared for the signal values of genes in the differential gene union. A series of expression tendency models of the differential genes in three groups were obtained. The minimum correlation coefficient of the state tendency analysis is 0.85. Significant models had a higher probability than expected by Fisher’s exact test and the multiple comparison tests [89].

### GO analysis and pathway analysis

Gene Ontology (GO) annotation of differential genes was based on the AmiGO database (http://amigo.geneontology.org/amigo). Using Fisher's exact test and χ^2^ test [89], we calculated the result of the multiple hypothesis testing correction to obtaining the FDR, which was produced to correct the P-value. The FDR was defined as FDR = 1- N_K_/T, where N_K_ refers to the number of Fisher’s test P-values less than the χ^2^ test P-values. Thus, the differentially expressed genes in significantly enriched GO terms were selected at P-value <0.01 and FDR <0.05. Pathway annotations of differential genes were downloaded from Kyoto Encyclopedia of Genes and Genomes (KEGG) (http://www.genome.jp/kegg/). A Fisher exact test was used to find the significant enrichment pathways. The resulting P-values were adjusted using the BH FDR algorithm [89]. Pathway categories with a P-value <0.05 were reported. Enrichment provided a measure of the significance of the function because more enrichment represents more significant pathways in the experiment.

### Gene-act network and co-expression network

We used the KEGG database to build the network of genes according to the relationship among the genes, proteins and compounds in the database [90–93]. Then, the gene co-expression networks were presented to find the interactions among the genes [94]. The gene co-expression networks were built according to the normalized signal intensity of specific expression genes. For each pair of genes, we calculated the Pearson correlation (P-value <0.05, Pearson >0.999 or <-0.999) and chose the significant correlation pairs to construct the network [95]. Within the network analysis, degree centrality is the simplest and most important measure of the centrality of a gene within a network to determine the relative importance. The degree centrality is defined as the number of links that one node has to other [96]. Moreover, to study the properties of the networks, k-cores in the graph theory were introduced as a method of simplifying the graph topology analysis. A k-core of a protein-protein interaction network usually contains cohesive groups of proteins [97, 98]. The purpose of network structure analysis was to locate the core regulatory factors (genes) that were in one network. Core regulatory factors connected most of the adjacent genes and had the largest degree. While considering different networks, the core regulatory factors were determined by the degree differences between two classes of samples. They usually had the largest degree of differences.

### QPCR validation

The reverse transcription of the three groups of extracted RNA was followed by PrimeScript RT Master Mix (TAKARA, Code No. RR036A). The reaction volume was 20 μL at the end and consisted of 4 μL 5 × PrimeScript RT Master Mix (Perfect Real Time), 1 μg total mRNA, and a supplement of nuclease-free water. The protocol of reverse transcription was 37°C for 15 min, and 85°C for 5 sec.

Twenty five genes were randomly selected to design specific primers with primer 6.0 (Table 6). The validation of RNA-Seq was conducted in a final volume of 20 μL that contained 3 μL of cDNA, 0.4 μM of each primer, 10 μL of ULtraSYBR Mixture and 6.2 μL of nuclease-free water. PCR amplifications were performed by the Bio-Rad CFX96 RealTime PCR system (Bio-Rad, US). The reaction program was the following: 95°C for 30 sec, 50 cycles (94°C for 15 sec, 58°C for 20 sec), and 72°C for 20 sec. The dissolution curve temperature is 60.0–95.0°C, which was increased by 0.5°C per 0.05 sec. The cDNA of each group with three parallels was normalized with the EF1A gene [99]. Then, the 2^-ΔΔCt^ method processing data was used to obtain the fold change [100].

**Table 6:** List of the genes validated by qPCR.

Gene	Abbreviation	Primer sequence	Amplicon length
Sulfotransferase family cytosolic 2B member 1	SULT2B1	GGTCAGCACACTTCAGCGATGAT	107
		GTCACTCTCATTCACTGGCATTGGA	
Glyceraldehyde-3-phosphate dehydrogenase	GAPDHS	AAGAAGCATCATCCGTGAGGTTACT	80
		TCCATTGATTCCAACACAGAGGTCT	
Complement C3	C3	GCAGCACCTGGTTGACAGCATAT	92
		GACGGCGTTACAGATCACTTCACTT	
cyclin-B2	CCNB2	TCTGAGGTCGCTGCTGCTTC	91
		CATAGGTAGAGTAGTGCTGCTGTGT	
D(4) dopamine receptor	DRD4	GGTTGCTTCTTGTTCTGTTGGACTC	101
		GTGACAGTGCTCATTAGACCAGGAT	
Small cytidine deaminase	CDA	GTGGAGAATGCGAGTTACAATCTGG	118
		GGTCACTCTCGTCACTGGCAAT	
fizzy-related protein homolog	FZR1	ATGCCTCCGCTCCAGTCAGA	111
		CTTGTTGTCATTGCCGCCAGAG	
Glutathione S-transferase theta-1	GSTT1	TCGCTCAACCTGTTGGAGGAGAA	94
		TCCACCACAGCCACCACATCT	
microtubule-associated protein tau	MAPT	GCAACTCGCAGGAGCATCTTCA	112
		CTTGGCGGTTATGGACGGTCTT	
Phosphatidate cytidylyltransferase 2	CDS2	CAAGGAGGCTGATGGCGATGAC	101
		TGGCGAGAGGATAATCTGGAGTGT	
Alpha-2-antiplasmin	SERPINF2	TCTGTTCTCTGGTCCTGACCTCTC	101
		CGACGCCTTCTTCACTGAGTTCTAT	
DNA replication licensing factor MCM3	MCM3	TGATTCAGAGGAGGAGGAGGAGATG	104
		CATACGGGCTGTAGGGCTCACT	
bile acid receptor	NR1H4	GGAATGCTGGCTGAATGTCTGTTGA	95
		CCTGCTGTCCGTGTTCTCCTCTT	
histone-lysine N-methyltransferase, H3 lysine-79 specific	DOT1L	AGGAGGTGAATCGGACGCAGAA	95
		ACGAAGAGGAAGACGAGGATGAAGA	
complement component C6	C6	GGACTCGCAGCATGGACCTT	100
		CAGCATCATCACGACTCACATTGG	
cyclin-dependent kinase 2	CDK2	GGCTGGACCATAGAGTCATTGAGAA	95
		CCACCTGCTGCCACCATCAT	
sonic hedgehog protein A	SHH	GGCAAGATCGCACGCAATTCTGA	108
		CGCCTGTGTTCTCCTCGTCCTT	
Diacylglycerol O-acyltransferase 2	DGAT2	CTAGGAAGATGACTGGTTGCTGCTA	91
		GTGCGATGACGAAGGTGGTGAA	
2-acylglycerol O-acyltransferase 2	MOGAT2	CTGCCACTTCACCATCTCTGTAACT	120
		TCAGCCAGTAGCGAGTGAACATC	
UDP-glucuronosyltransferase 2B17	UGT2B17	TCAGATTGCCCAGTATTTGCTACCA	113
		CCACTCTCATTAGCCACAGGTCAG	
cDNA, FLJ96434, highly similar to Homo sapiens cytochrome P450, family 2, subfamily J, polypeptide 2 (CYP2J2), mRNA	CYP2J2	ACTTTGCTGGGACTGACACTACATC	102
		GGTCTATCTCCTCCTGGCATTGTTC	
ITGA10 protein	ITGA10	GGCACCACCATATCCAGTCTTCCA	95
		ACGGCACCTCTGTTATCATCCTCAA	
3-keto-steroid reductase	HSD17B7	CCTTACACCGTACAATGGAGCAGAG	108
		GTCCCAAGCCCTGATGTTAAACTGT	
UDP-glucuronosyltransferase 2A1	UGT2A1	CACACCTTGTATAACGCAACTCAGT	92
		GCTTCAAGTTCGCTATCACGGTTA	
cDNA FLJ75329, highly similar to Homo sapiens LAG1 longevity assurance homolog 2 (S. cerevisiae), transcript variant 2, mRNA	CERS2	CGGTTGATGACAATGATGAAGAGGA	95
		GTTAGCCAGCGATGCCAGTCT	
Patatin-like phospholipase domain containing 2	PNPLA2	GGCTGGACCATAGAGTCATTGAGAA	95
		CCACCTGCTGCCACCATCAT	
cDNA FLJ75392, highly similar to Homo sapiens hexokinase II (HKII) mRNA	HK2	GGCGAGTCCAACATTCAGATAACCA	107
		TACTTCTCCACCTTACCGAGCACAT	

## Supporting Information

S1 Fig Responses of the steroid biosynthesis pathway to salinity acclimation in *O*. *niloticus*. The steroid biosynthesis pathway is up-regulated in the constant-change category. The quadrilateral in red represents the up-regulated genes. The 2.5.1.21 represents the squalene synthase; the 1.14.13.132 represents the squalene monooxygenase; the 1.3.1.72 represents the delta24-sterol reductase and the 1.1.1.270 represents the 3-keto steroid reductase. According to this pathway, the production of cholesterol is up-regulated.
(TIF)

S2 Fig Responses of the ovarian steroidogenesis pathway to salinity acclimation in *O*. *niloticus*. The ovarian steroidogenesis pathway is up-regulated both involved in the constant-change and stable-then-change categories. The quadrilateral in red represents the up-regulated genes. The IGF1R represents the insulin-like growth factor 1 receptor; the AC represents the adenylate cyclase 1; the ARTISt represents the acyl-CoA thioesterase 2; the LPOX represents the arachidonate 5-lipoxygenase; the CYP17 represents the steroid 17 alpha-monooxygenase / 17 alpha-hydroxyprogesterone aldolase and the 17β-HSD represents the 17 beta-estradiol 17-dehydrogenase. According to this pathway, ovarian steroidogenesis participates in the cAMP signal pathway regulation.
(TIF)

S3 Fig Responses of the steroid hormone biosynthesis pathway to salinity acclimation in *O*. *niloticus*. The steroid hormone biosynthesis pathway is up-regulated in the constant-change category. The quadrilateral in red represents the up-regulated gene. The 2.8.2.2 represents the alcohol sulfotransferase; the 1.1.1.62 represents the 17 beta-estradiol 17-dehydrogenase and the 2.4.1.17 represents the glucuronosyltransferase. According to this pathway, many downstream steroid hormones are up-regulated under salinity stress.
(TIF)

S4 Fig PPAR signaling pathway response to salinity acclimation in *O*. *niloticus*. The PPAR signaling pathway is down-regulated in the change-then-stable category. The quadrilateral in blue represents the down-regulated gene. The FABP represents the fatty acid-binding protein 1; the PPARα represents the peroxisome proliferator-activated receptor alpha; the RXR represents the retinoid X receptor alpha; the Apo-AI, Apo-AⅡand Apo-AⅤrepresent the apolipoprotein A family; and the ACS represents the long-chain acyl-CoA synthetase.
(TIF)

S5 Fig Fat digestion and absorption pathway response to salinity acclimation in *O*. *niloticus*. The fat digestion and absorption pathway is down-regulated in the constant-change and change-then-stable categories. The quadrilateral in blue represents the down-regulated gene. The I-FABP represents the fatty acid-binding protein 2; the MGAT represents the 2-acylglycerol O-acyltransferase 2; the DGAT represents the diacylglycerol O-acyltransferase 1; the PAP represents the phosphatidate phosphatase; the ApoB-48 represents the apolipoprotein B; the ApoA-IV represents the apolipoprotein A-IV and the ApoA-Ⅰrepresents the apolipoprotein A-I. In this pathway, down-regulated genes in triglycerides biosynthesis and transportation indicate that triglyceride utilization is up-regulated under salinity stress.
(TIF)

S6 Fig Responses of the fatty acid biosynthesis pathway to salinity acclimation in *O*. *niloticus*. The fatty acid biosynthesis pathway is down-regulated in the change-then-stable category. The quadrilateral in blue represents the down-regulated gene. The FASN represents the fatty acid synthase. The down regulation of fatty acid synthase suggests that the acetyl-CoA is reserved to participate in physical synthesis and energy production.
(TIF)

S7 Fig Biosynthesis of unsaturated fatty acids pathway response to salinity acclimation in *O*. *niloticus*. The biosynthesis of unsaturated fatty acids pathway is up-regulated in the stable-then-change category. The quadrilateral in red represents the up-regulated gene. The 3.1.2.2 represents the acyl-coenzyme A thioesterase. Because the contents of unsaturated fatty acids in feed cannot meet the demands in response to ambient salinity, Nile tilapia have to synthesis by themselves.
(TIF)

S8 Fig Glycolysis/Gluconeogenesis pathway response to salinity acclimation in *O*. *niloticus*. The glycolysis/gluconeogenesis pathway is up-regulated in the stable-then-change category. The quadrilateral in red represents the up-regulated gene. The 2.7.1.1 represents the hexokinase; the 4.1.2.13 represents the fructose-bisphosphate aldolase; the 5.3.1.1 represents the triosephosphate isomerase; the 5.4.2.11 represents the 2,3-bisphosphoglycerate-dependent phosphoglycerate mutase; the 4.1.1.32 represents the phosphoenolpyruvate carboxykinase; the 1.2.1.3 represents the aldehyde dehydrogenase; the 6.2.1.1 represents the acetyl-CoA synthetase; the 2.7.1.40 represents the pyruvate kinase and the 1.1.1.27 represents the L-lactate dehydrogenase. Even the restriction enzymes, pyruvate kinase and hexokinase, are up-regulated, the 6-phosphofructokinase remains unchanged. Therefore, it is difficult to access the glycolytic efficiency.
(TIF)

S9 Fig Pyruvate metabolism pathway response to salinity acclimation in *O*. *niloticus*. The Pyruvate metabolism pathway is up-regulated in the stable-then-change category. The quadrilateral in red represents the up-regulated gene. The 4.1.1.32 represents the phosphoenolpyruvate carboxykinase; the 2.7.1.40 represents the pyruvate kinase; the 1.1.1.28 represents the D-lactate dehydrogenase and the 6.2.1.1 represents the acetyl-CoA synthetase.
(TIF)

S10 Fig The detection results of the mixed total RNA quality on a Bioanalyzer 2200. A0 represents ladder; A1/AL represents the freshwater treated set; the B1/BL represents the 8 psu treated set and the C1/CL represents the 16 psu treated set. The RIN^e^ value >8 is acceptable for cDNA library construction.
(TIF)

S1 Table The whole body crude compositions of Nile tilapia at different salinity acclimations (%). (DOCX)

S2 Table The growth parameters of Nile tilapia at different salinity acclimations. (DOCX)

S3 Table The compositions of commercial diet used in the study. (DOCX)
