# Supplementary figures and images for: Transcriptome Profiling and Molecular Pathway Analysis of Genes in Association with Salinity Adaptation in Nile Tilapia Oreochromis niloticus
Source: PLoS One. 2015 Aug 25;10(8):e0136506. doi: 10.1371/journal.pone.0136506 (PMC4548949; doi:10.1371/journal.pone.0136506)

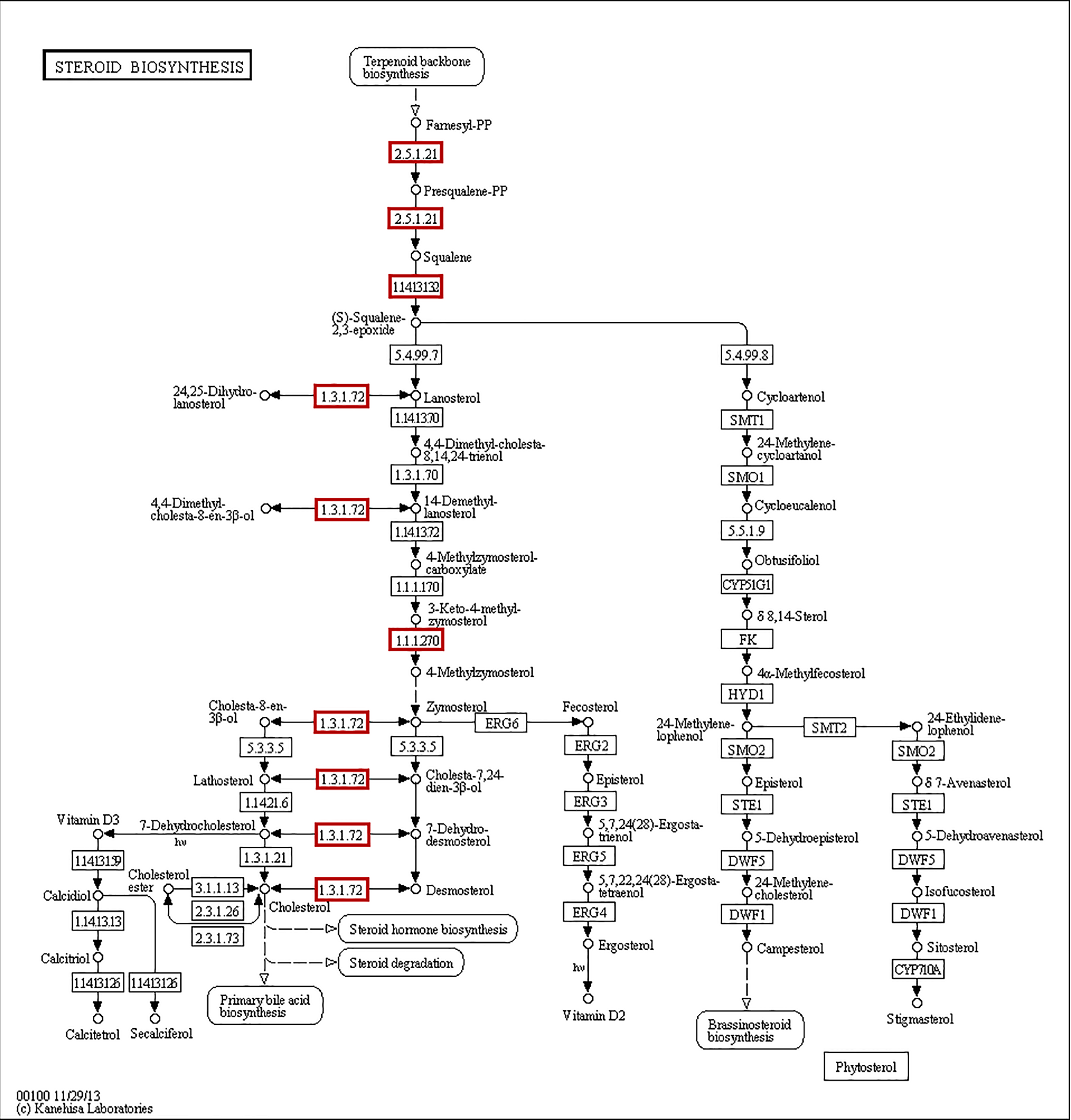

Supplement: S1 Fig — The steroid biosynthesis pathway is up-regulated in the constant-change category. The quadrilateral in red represents the up-regulated genes. The 2.5.1.21 represents the squalene synthase; the 1.14.13.132 represents the squalene monooxygenase; the 1.3.1.72 represents the delta24-sterol reductase and the 1.1.1.270 represents the 3-keto steroid reductase. According to this pathway, the production of cholesterol is up-regulated. (TIF) [file pone.0136506.s001.tif]

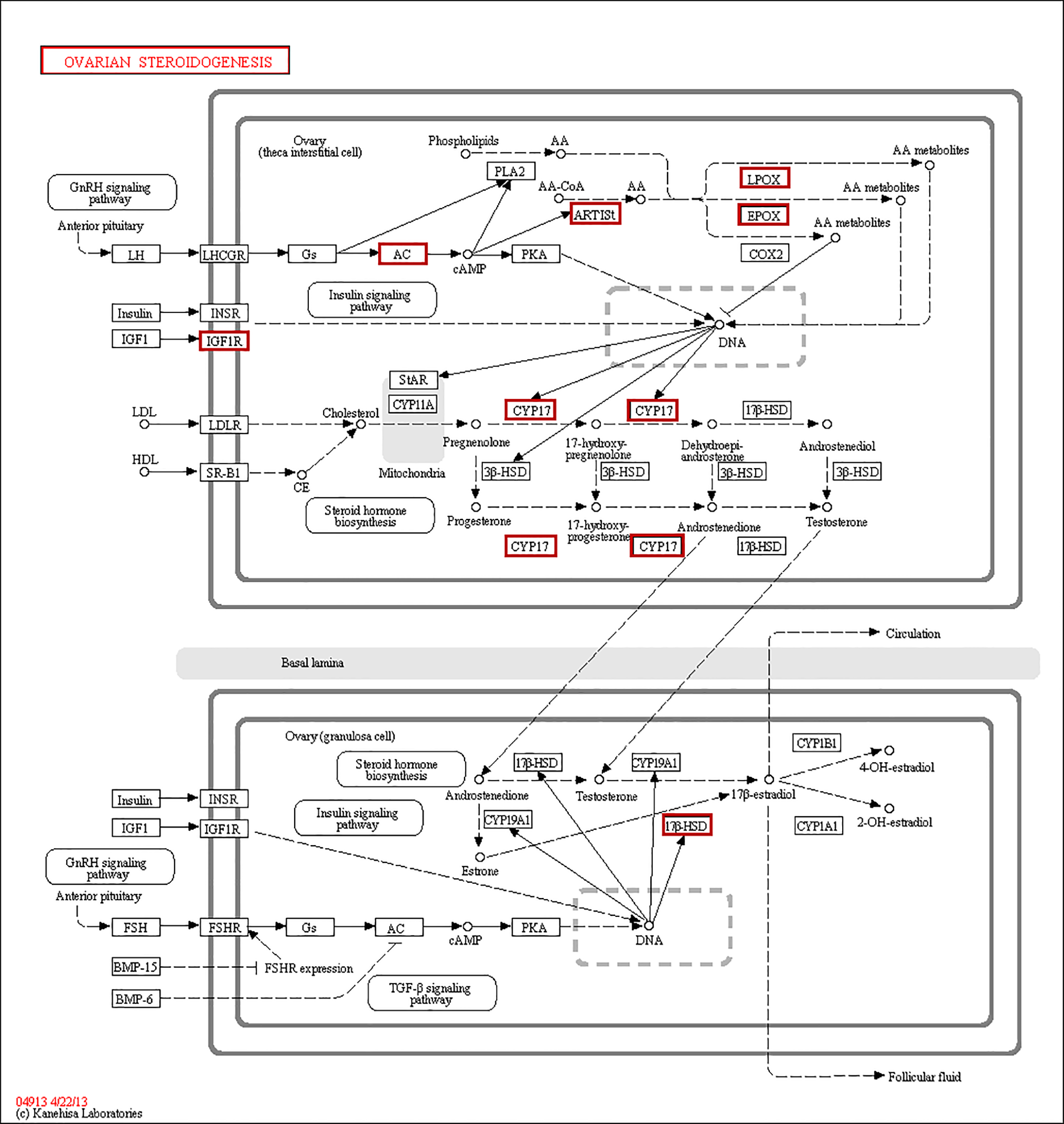

Supplement: S2 Fig — The ovarian steroidogenesis pathway is up-regulated both involved in the constant-change and stable-then-change categories. The quadrilateral in red represents the up-regulated genes. The IGF1R represents the insulin-like growth factor 1 receptor; the AC represents the adenylate cyclase 1; the ARTISt represents the acyl-CoA thioesterase 2; the LPOX represents the arachidonate 5-lipoxygenase; the CYP17 represents the steroid 17 alpha-monooxygenase / 17 alpha-hydroxyprogesterone aldolase and the 17β-HSD represents the 17 beta-estradiol 17-dehydrogenase. According to this pathway, ovarian steroidogenesis participates in the cAMP signal pathway regulation. (TIF) [file pone.0136506.s002.tif]

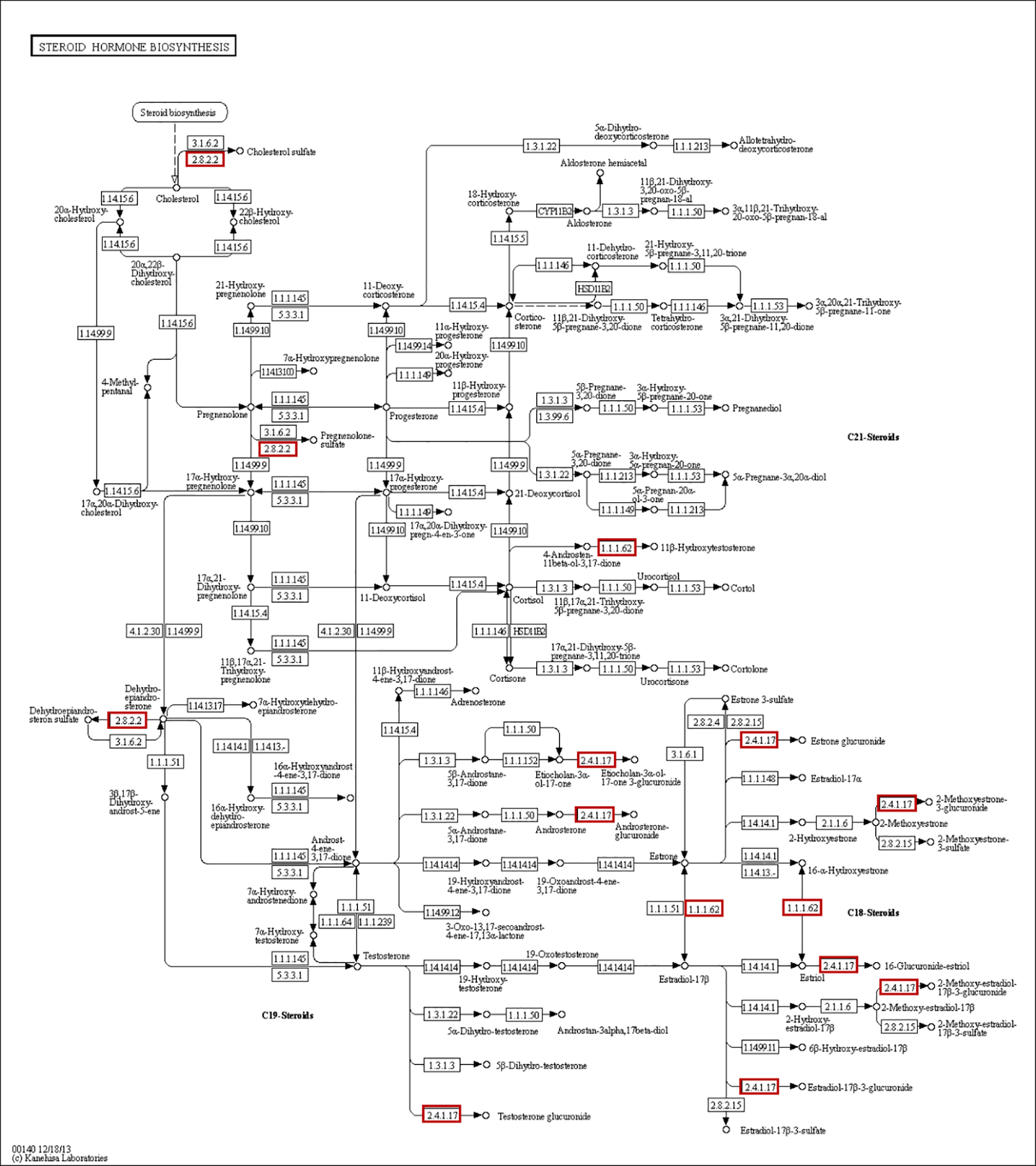

Supplement: S3 Fig — The steroid hormone biosynthesis pathway is up-regulated in the constant-change category. The quadrilateral in red represents the up-regulated gene. The 2.8.2.2 represents the alcohol sulfotransferase; the 1.1.1.62 represents the 17 beta-estradiol 17-dehydrogenase and the 2.4.1.17 represents the glucuronosyltransferase. According to this pathway, many downstream steroid hormones are up-regulated under salinity stress. (TIF) [file pone.0136506.s003.tif]

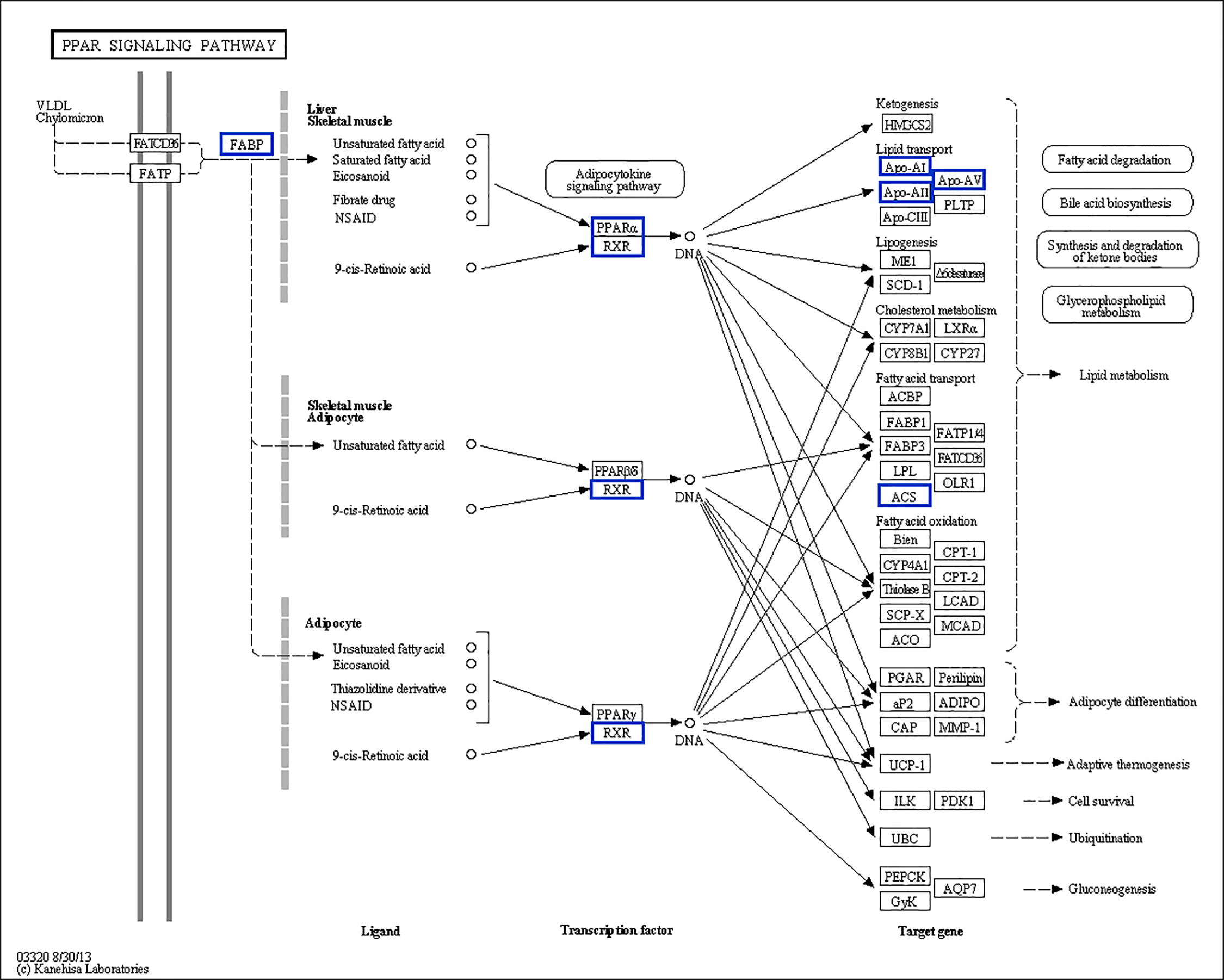

Supplement: S4 Fig — The PPAR signaling pathway is down-regulated in the change-then-stable category. The quadrilateral in blue represents the down-regulated gene. The FABP represents the fatty acid-binding protein 1; the PPARα represents the peroxisome proliferator-activated receptor alpha; the RXR represents the retinoid X receptor alpha; the Apo-AI, Apo-AⅡand Apo-AⅤrepresent the apolipoprotein A family; and the ACS represents the long-chain acyl-CoA synthetase. (TIF) [file pone.0136506.s004.tif]

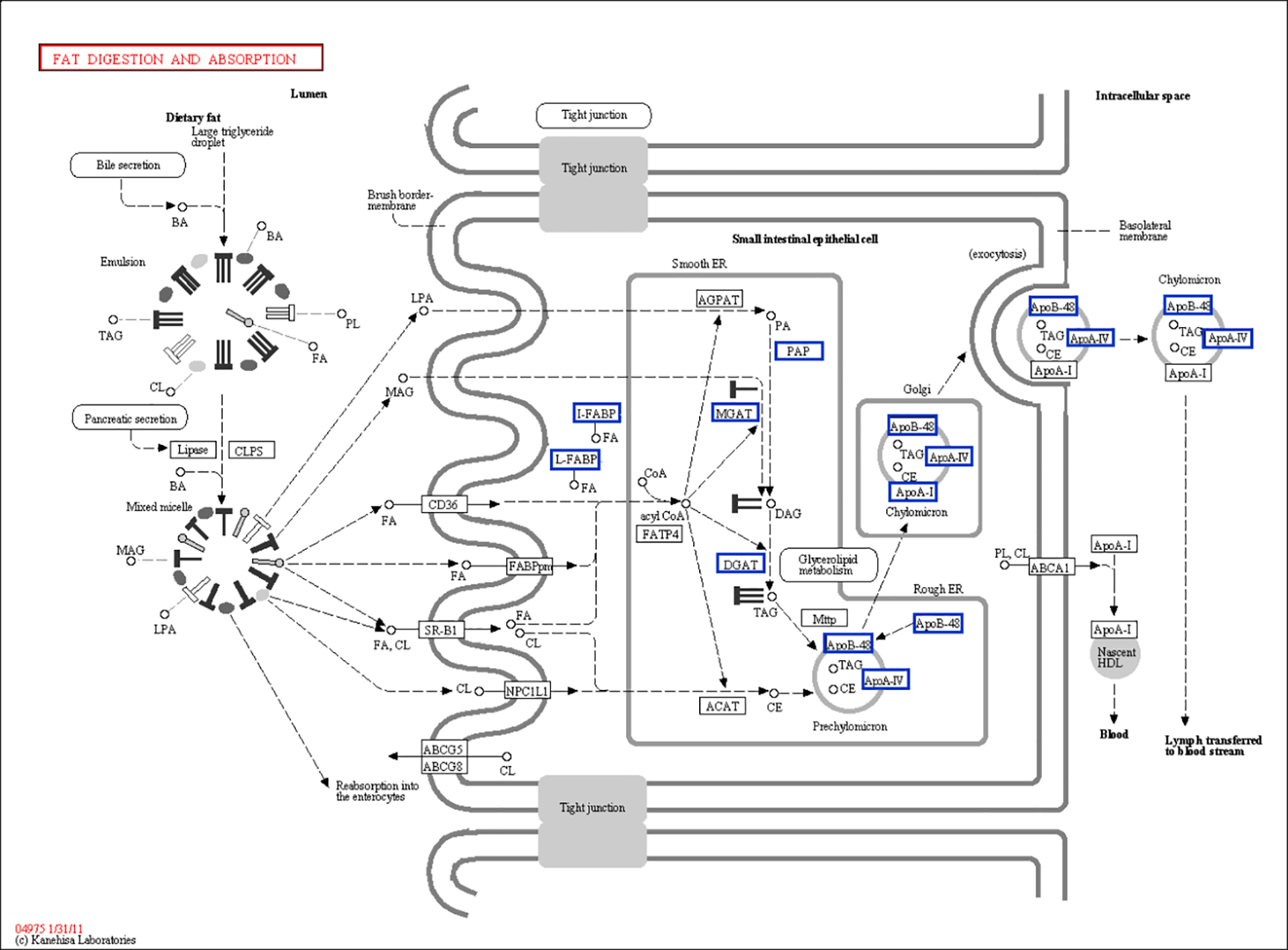

Supplement: S5 Fig — The fat digestion and absorption pathway is down-regulated in the constant-change and change-then-stable categories. The quadrilateral in blue represents the down-regulated gene. The I-FABP represents the fatty acid-binding protein 2; the MGAT represents the 2-acylglycerol O-acyltransferase 2; the DGAT represents the diacylglycerol O-acyltransferase 1; the PAP represents the phosphatidate phosphatase; the ApoB-48 represents the apolipoprotein B; the ApoA-IV represents the apolipoprotein A-IV and the ApoA-Ⅰrepresents the apolipoprotein A-I. In this pathway, down-regulated genes in triglycerides biosynthesis and transportation indicate that triglyceride utilization is up-regulated under salinity stress. (TIF) [file pone.0136506.s005.tif]

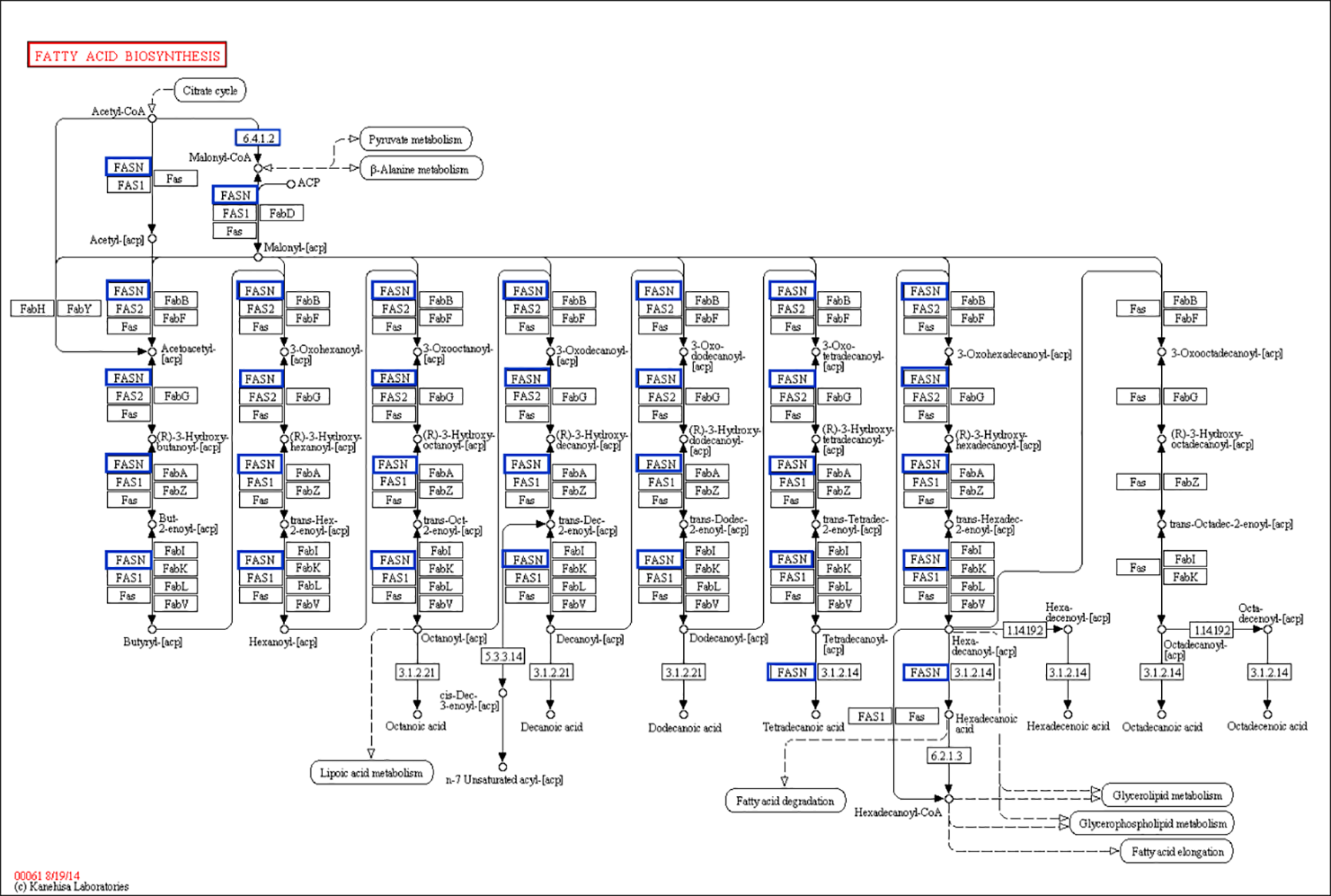

Supplement: S6 Fig — The fatty acid biosynthesis pathway is down-regulated in the change-then-stable category. The quadrilateral in blue represents the down-regulated gene. The FASN represents the fatty acid synthase. The down regulation of fatty acid synthase suggests that the acetyl-CoA is reserved to participate in physical synthesis and energy production. (TIF) [file pone.0136506.s006.tif]

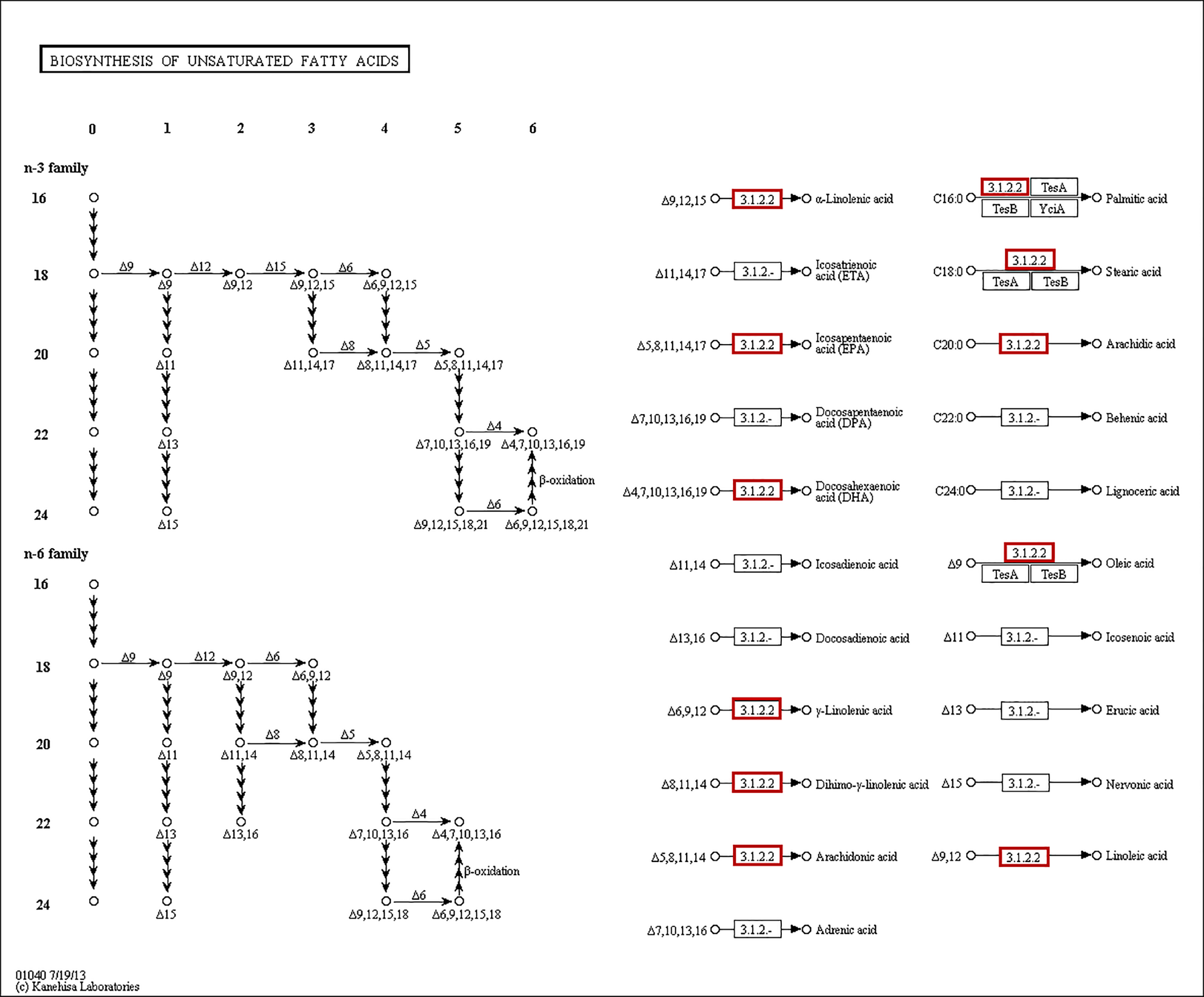

Supplement: S7 Fig — The biosynthesis of unsaturated fatty acids pathway is up-regulated in the stable-then-change category. The quadrilateral in red represents the up-regulated gene. The 3.1.2.2 represents the acyl-coenzyme A thioesterase. Because the contents of unsaturated fatty acids in feed cannot meet the demands in response to ambient salinity, Nile tilapia have to synthesis by themselves. (TIF) [file pone.0136506.s007.tif]

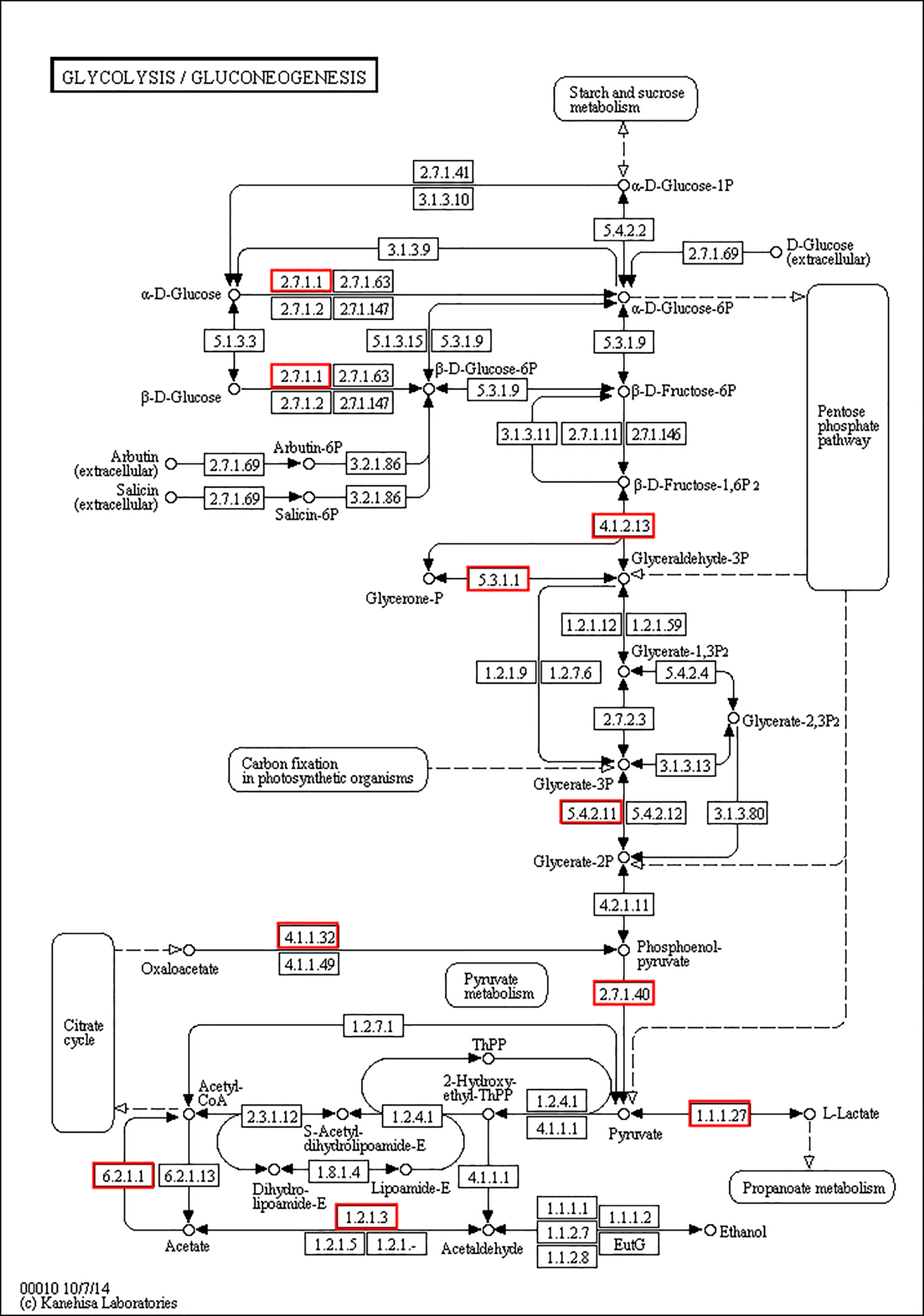

Supplement: S8 Fig — The glycolysis/gluconeogenesis pathway is up-regulated in the stable-then-change category. The quadrilateral in red represents the up-regulated gene. The 2.7.1.1 represents the hexokinase; the 4.1.2.13 represents the fructose-bisphosphate aldolase; the 5.3.1.1 represents the triosephosphate isomerase; the 5.4.2.11 represents the 2,3-bisphosphoglycerate-dependent phosphoglycerate mutase; the 4.1.1.32 represents the phosphoenolpyruvate carboxykinase; the 1.2.1.3 represents the aldehyde dehydrogenase; the 6.2.1.1 represents the acetyl-CoA synthetase; the 2.7.1.40 represents the pyruvate kinase and the 1.1.1.27 represents the L-lactate dehydrogenase. Even the restriction enzymes, pyruvate kinase and hexokinase, are up-regulated, the 6-phosphofructokinase remains unchanged. Therefore, it is difficult to access the glycolytic efficiency. (TIF) [file pone.0136506.s008.tif]

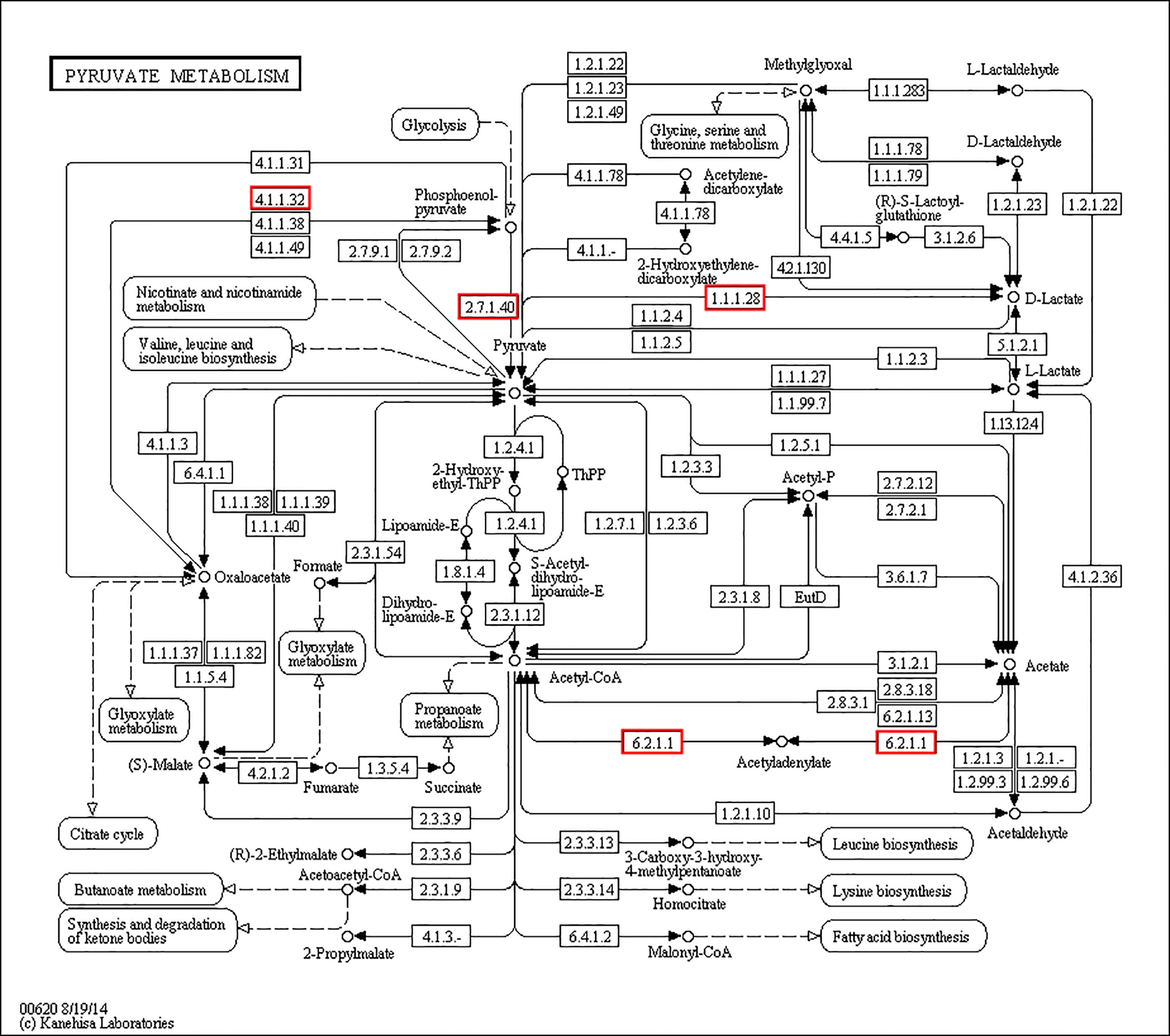

Supplement: S9 Fig — The Pyruvate metabolism pathway is up-regulated in the stable-then-change category. The quadrilateral in red represents the up-regulated gene. The 4.1.1.32 represents the phosphoenolpyruvate carboxykinase; the 2.7.1.40 represents the pyruvate kinase; the 1.1.1.28 represents the D-lactate dehydrogenase and the 6.2.1.1 represents the acetyl-CoA synthetase. (TIF) [file pone.0136506.s009.tif]

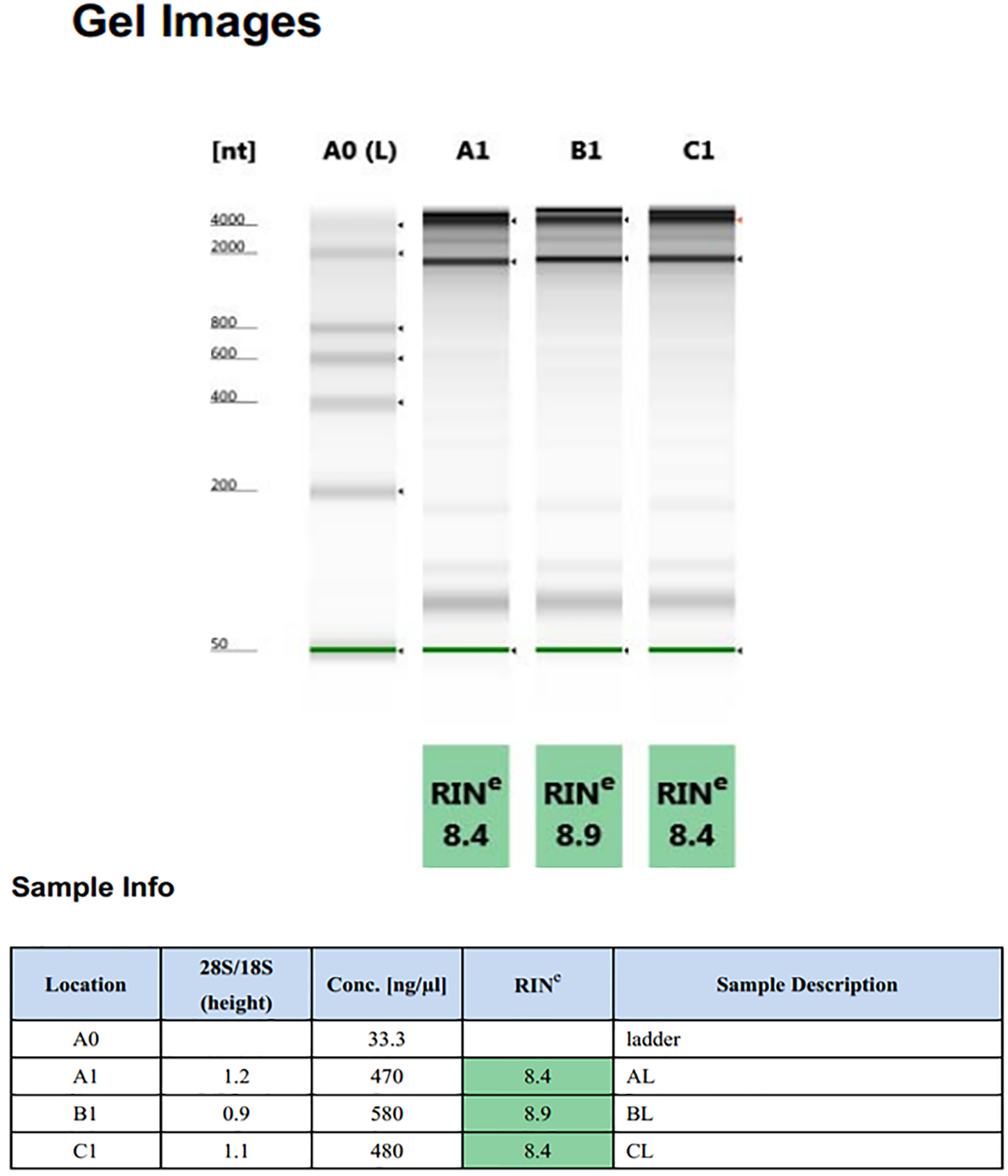

Supplement: S10 Fig — A0 represents ladder; A1/AL represents the freshwater treated set; the B1/BL represents the 8 psu treated set and the C1/CL represents the 16 psu treated set. The RINe value >8 is acceptable for cDNA library construction. (TIF) [file pone.0136506.s010.tif]
