# Supplementary material for: Transcriptome Profiling and Molecular Pathway Analysis of Genes in Association with Salinity Adaptation in Nile Tilapia Oreochromis niloticus
Source: PLoS One. 2015 Aug 25;10(8):e0136506. doi: 10.1371/journal.pone.0136506 (PMC4548949; doi:10.1371/journal.pone.0136506)
Supplement: S1 Table — (DOCX) [file pone.0136506.s011.docx]

**S1 Table**

The whole body crude compositions of Nile tilapia at different salinity acclimations (%)

| Composition | Group | | |
| --- | --- | --- | --- |
|  | Control | 8 psu | 16 psu |
| Moisture | 72.73±1.49 | 75.17±1.44 | 74.35±1.57 |
| Crude lipid | 8.89±0.86^a^ | 8.35±0.86^a^ | 6.41±0.51^b^ |
| Crude protein | 11.03±0.66 | 10.15±0.34 | 10.39±0.54 |
| Ash | 2.62±0.15 | 2.57±0.12 | 2.65±0.11 |

Values: mean ± SD (n = 3/group). Values in the same row with different superscripts are statistically significant differences (p<0.05).

There were no significant difference at moisture, crude protein and ash. Crude lipid was the significantly different composition which supported the conclusion of lipid consumption of RNA-Seq results of *O. niloticus* at saline water.
