# Supplementary material for: Transcriptome Profiling and Molecular Pathway Analysis of Genes in Association with Salinity Adaptation in Nile Tilapia Oreochromis niloticus
Source: PLoS One. 2015 Aug 25;10(8):e0136506. doi: 10.1371/journal.pone.0136506 (PMC4548949; doi:10.1371/journal.pone.0136506)
Supplement: S2 Table — (DOCX) [file pone.0136506.s012.docx]

**S2 Table**

The growth parameters of Nile tilapia at different salinity acclimations

| Parameter | Group | | |
| --- | --- | --- | --- |
|  | Control | 8 psu | 16 psu |
| Weight gain | 1143.70±410.54^a^ | 762.38±161.56^ab^ | 512.12±200.07^b^ |
| Survival | 84.00±12.00 | 86.67±8.33 | 86.67±6.11 |
| FCR | 1.54±0.57^a^ | 2.13±0.12^ab^ | 2.85±0.58^b^ |
| HSI(%) | 2.19±0.22 | 2.27±0.19 | 2.07±0.08 |
| CF | 3.52±0.28 | 3.59±0.20 | 3.50±0.23 |

Values: mean ± SD (n=3/group). Values in the same row with different superscripts are statistically significant differences (p<0.05).

These were main parameters of *O. niloticus* reared at different salinities. The significant influence of different salinities on *O. niloticus* was weight gain. No significant difference was found in survival, FCR, HSI and CF. The results showed that it is feasible for *O. niloticus* rearing at saline water because there was no significant difference in survival. But significant difference in weight gain showed the *O. niloticus* was under stress at saline water.
