# Supplementary material for: Transcriptome Profiling and Molecular Pathway Analysis of Genes in Association with Salinity Adaptation in Nile Tilapia Oreochromis niloticus
Source: PLoS One. 2015 Aug 25;10(8):e0136506. doi: 10.1371/journal.pone.0136506 (PMC4548949; doi:10.1371/journal.pone.0136506)
Supplement: S3 Table — (DOCX) [file pone.0136506.s013.docx]

**S3 Table**

The compositions of commercial diet used in the study

| Crude protein≥% | crude fat≥% | crude fiber≤% | Crude ash≤% | calcium≤% | Total scales≤% | Water content≤% | Total lysine≥% |
| --- | --- | --- | --- | --- | --- | --- | --- |
|  |  |  |  |  |  |  |  |
| 35 | 3 | 9 | 13 | 0.9-1.3 | 0.75 | 12 | 1.5 |

The commercial diet was produced by Shandong Dongpinghu Feed Co., Ltd. China. Feed ingredient：fish meal, wheat flour, FD shrimp powder, soya protein, germ, brewer's yeast, seaweed powder, starch, vegetable oils, digestive enzymes, amino acids, carotenoids, multiple vitamins, mineral nutrients, and stable vitamin C.
